# Reconfigurable In–S Coordination in SPAN Cathodes: Unlocking High Sulfur Utilization and Fast Kinetics for Practical Li‒S Batteries

**DOI:** 10.1002/advs.202507385

**Published:** 2025-07-30

**Authors:** Cheng Huang, Yi Gong, Qi Zhu, Miaoran Xu, Kai Yang, José V. Anguita, Wei Zhang, S. Ravi P. Silva, Yanfeng Gao, Zongtao Zhang

**Affiliations:** ^1^ School of Materials Science and Engineering Zhengzhou University Zhengzhou 450001 China; ^2^ School of Computer Science and Electronic Engineering University of Surrey Guildford Surrey GU2 7XH UK; ^3^ School of Materials Science and Engineering Shanghai University Shanghai 200444 China

**Keywords:** amorphous indium‐sulfur composite, high sulfur loading, Li‒S batteries, redox kinetics, sulfurized polyacrylonitrile

## Abstract

Sulfurized polyacrylonitrile (SPAN) has emerged as a promising cathode material for high‐energy‐density lithium‒sulfur (Li‒S) batteries due to its ability to confine sulfur and suppress polysulfide shuttling. However, conventional SPAN suffers from sluggish conversion kinetics and limited sulfur utilization, especially at high sulfur loadings. In this work, reconfigurable indium‒sulfur (In–S) coordination into SPAN to dynamically regulate sulfur bonding states is introduced. The non‐crystalline In—S network reversibly anchors and releases sulfur during cycling, accelerating redox reactions while suppressing phase segregation. Structural analysis reveals atomically dispersed In—S coordination without crystalline inactive phases, achieving an active material content of 47.4 wt.% with only 1.18 wt.% indium addition (≈23% higher than conventional SPAN). Optimized In_5_‐SPAN cathodes deliver a high specific capacity of 1048 mAh·g^−1^ at 0.5 A g^−1^ under practical conditions of high SPAN mass loading (8.7 mg cm^−2^) and lean electrolyte (E/SPAN = 4.1). This performance surpasses state‐of‐the‐art SPAN‐based cathodes under comparable lean‐electrolyte and high‐loading conditions. These findings illustrate a novel reconfigurable metal‒sulfur coordination strategy for next‐generation Li‒S batteries with both high‐energy‐density and long cycle life.

## Graphical abstract

An amorphous indium–sulfur complex is embedded in SPAN matrix to create reversible In–S coordination bonds that dynamically break and reform during cycling. This bond reconfiguration actively anchors intermediate polysulfides and accelerates sulfur conversion kinetics, providing simultaneously high active material utilization and durable cycling performance in lithium–sulfur batteries.

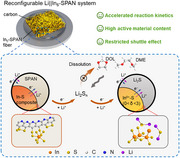

## 1 Introduction

The rapidly growing demand for high‐energy‐density and low‐cost energy storage systems poses a significant challenge to the current lithium‐ion batteries, which are approaching their theoretical limits.^[^ ^1^ ^]^ Lithium‒sulfur (Li‒S) batteries, with their exceptionally theoretical specific capacity (1675 mAh·g^−1^), abundant sulfur reserves, and low cost, have garnered considerable attention as a promising candidate for next‐generation energy storage systems.^[^ ^2^ ^]^ Nevertheless, the practical application of Li‐S batteries is hindered by several challenges, including long‐chain polysulfide shuttling, limited reaction kinetics, and drastic volume changes during cycling.^[^ ^3^ ^]^ Notably, the dissolution and shuttling of high‐order Li_2_S_n_ (n≥ 4) can be circumvented by using sulfurized polyacrylonitrile (SPAN) as the cathode, which constrains sulfur species through covalent bonding and enables solid‐state conversion pathways that avoid polysulfide dissolution.^[^ ^4^ ^]^ These systems demonstrate nearly 100% Coulombic efficiency and remarkable cycling stability, making the SPAN cathode a promising and potentially low‐cost alternative for sulfur‐based batteries.^[^ ^5^ ^]^

Despite these advantages, fundamental limitations persist in the current SPAN architecture. The rigid cyclization of PAN during synthesis restricts accessible sulfur anchoring sites, limiting the sulfur content to below ≈40 wt.%. Moreover, inherently sluggish solid–solid conversions among SPAN, short‐chain lithium polysulfides (Li_2_S_n_, n≤4), and Li_2_S lead to low ionic/electronic conductivity, resulting in poor reaction kinetics and diminished rate performance.^[^ ^6^ ^]^ To address these challenges, various strategies, such as heteroatomic doping, catalytic additives, and conductive carbon incorporation, have been employed to enhance the reaction kinetics and cycling durability of SPAN.^[^ ^7^, ^8^, ^9^ ^]^ For example, incorporating iodine,^[^ ^10^ ^]^ CoS_2_,^[^ ^11^ ^]^ Fe_1‐x_S,^[^ ^12^ ^]^ and Co_10_‐SPAN‐CNT,^[^ ^13^ ^]^ have been shown to facilitate charge‐transfer or mediate redox reaction, thereby accelerating sulfur utilization and improving kinetics. However, a critical trade‐off persists. Because these dopants are electrochemically inert within the Li‒S voltage window, they do not participate in the sulfur redox process, and their presence irreversibly displaces a portion of active sulfur, compromising the overall gravimetric energy density.^[^ ^14^ ^]^ Recent studies have found that incorporating reversible Se─S or Te─S bonds can accelerate kinetics while maintaining a high sulfur content.^[^ ^6^, ^7^ ^]^ Such dynamically tunable design creates self‐optimizing systems that synergistically improve both active material content and redox kinetics.

Emerging breakthroughs in amorphous metal polysulfide chemistry offer new design principles for Li‒S cathodes.^[^ ^15^ ^]^ Unlike rigid crystalline chalcogenides, amorphous matrices provide flexible, anisotropic coordination environments that accommodate dynamic bond reconfiguration during redox processes. These amorphous hosts can also strongly adsorb polysulfides and accelerate the reaction kinetics.^[^ ^16^ ^]^ Indium sulfides are especially attractive due to their multifunctionality: the In^3+^/In^0^ redox couple lies within the Li‒S operating voltage window (1.0‒3.0 V vs Li/Li⁺).^[^ ^17^ ^]^ Studies have shown that a dynamically formed LiInS_2_ phase can act as a bidirectional redox mediator, significantly lowering the activation energy for both Li_2_S_n_ reduction (Li_2_S_n_ to Li_2_S) and Li_2_S oxidization (Li_2_S to Li_2_S_n_).^[^ ^3^, ^18^ ^]^ Meanwhile, defective In_2_S_3‐x_ exhibits enhanced polysulfide adsorption due to abundant active sites.^[^ ^19^ ^]^ Moreover, partial dissolution of indium species in the electrolyte can create in situ Li‒In interphases on the lithium anode, which improves cycling stabilities by protecting the anode.^[^ ^3^, ^18^, ^20^ ^]^

In this work, we synthesize an amorphous indium‐sulfur complex embedded in SPAN (denoted In_x_‐SPAN, x = 0, 2.5, 5, and 10) via electrospinning followed by sulfurization. Owing to the strong sulfur‐coordinating ability of indium (In) atoms, the optimized In_5_‐SPAN achieves an active material content of 47.4 wt.% with only 1.18 wt.% In. The amorphous indium‐sulfur complex undergoes a dynamic and reversible transformation between In–S and In^δ+^(0<δ<3) states, creating numerous coordinatively unsaturated In–S sites that lower reaction polarization and energy barriers, and thereby accelerating the reaction kinetics. Consequently, Li‐SPAN cells with In_5_‐SPAN cathodes exhibit outstanding high‐rate performance (delivering 1034 mAh·g^−1^ at 4 A g^−1^) and a high capacity of 1048 mAh·g^−1^ at 0.5 A g^−1^ under practical conditions (8.7 mg cm^−2^ SPAN loading and lean electrolyte condition with E/SPAN = 4.1). This strategy of dynamically modulating metal–sulfur bonds provides a new perspective for concurrently maximizing the active sulfur content and boosting the redox reaction kinetics in Li–S batteries.

## 2 Results and Discussion

**Figure 1:**
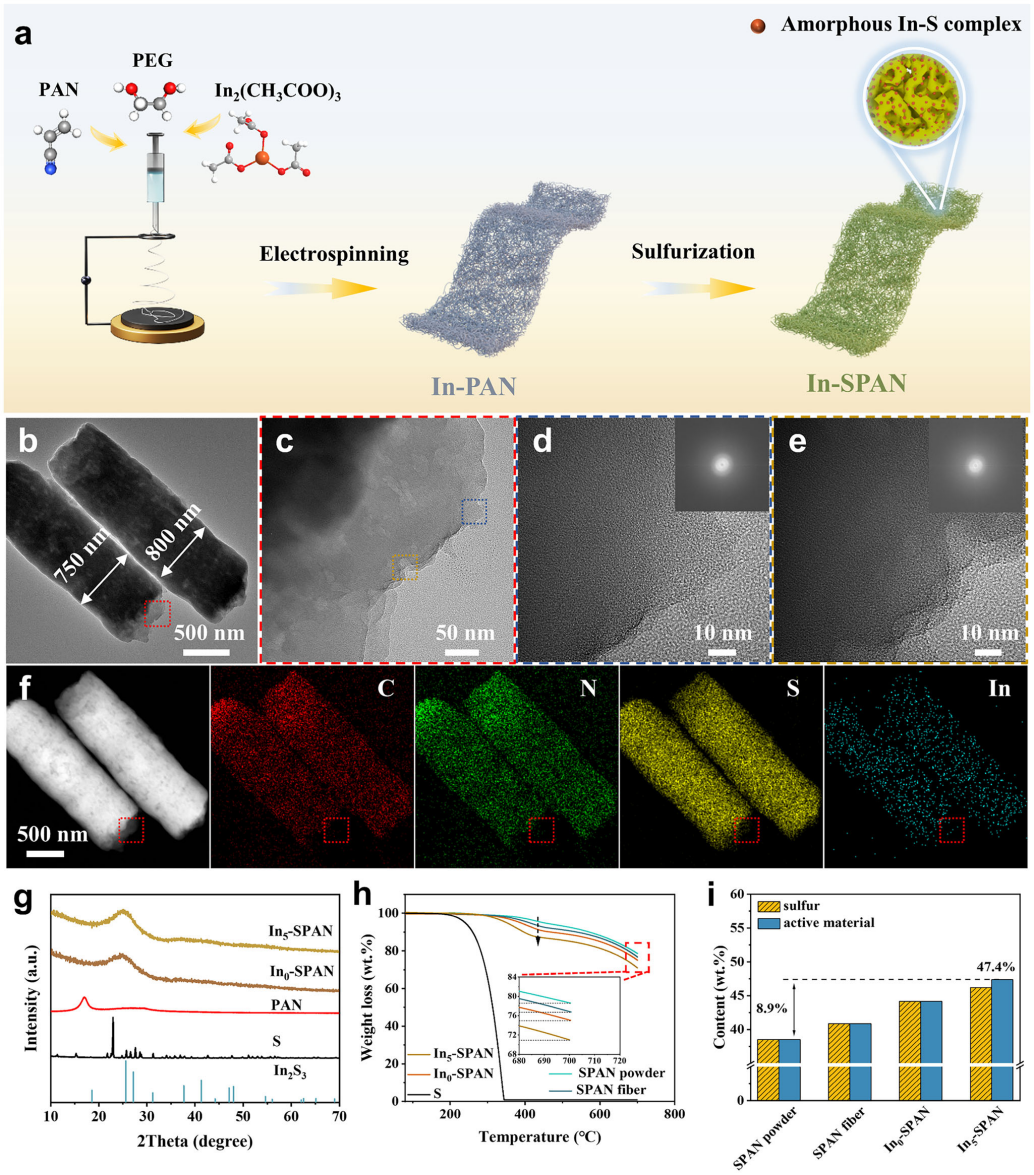
Synthesis and characterization of In_x_‐SPAN composites. a) Schematic synthesis process of In_5_‐SPAN cathode material. b‐c) TEM and d‐e) high‐resolution TEM (HRTEM) images of In_5_‐SPAN. Corresponding selected area electron diffraction (SAED) patterns from blue and brown rectangular regions in c) are shown as insets. f) Dark‐field TEM image of figure b, and corresponding elemental mapping images of In_5_‐SPAN. g) XRD patterns of In_5_‐SPAN, In_0_‐SPAN, PAN, S, and In_2_S_3_ (JCPDS: 71–1813), respectively. h) Thermogravimetric (TG) analysis (Inset shows magnified region indicated by a red rectangle and i) sulfur or active material content comparison of various materials.

The In_x_‐SPAN cathodes are prepared via a two‐step process of electrospinning and sulfurization (**Figure** **1**a). During the annealing sulfurization, In_2_(CH_3_COO)_3_ and PAN react with elemental sulfur to form SPAN copolymers integrated with amorphous In–S composites. The as‐prepared nanofiber mat (In_5_‐SPAN shown in Figure 1b; Figure S1a, Supporting Information) consists of uniform fibers with interconnected micropores of 700–900 nm diameter. Scanning electron microscopy (SEM) cross‐sectional images of individual fibers (Figure S1b, Supporting Information) and transmission electron microscopy (TEM) images confirm the presence of hierarchical nano‐ and micro‐pores created by the decomposition of the added PEG polymer. This porosity leads to a substantial increase in the Brunner‐Emmet‐Teller (BET) surface area from 8.1 m^2^ g^−1^ (for control without PEG) to 22.1 m^2^ g^−1^ with PEG (Figure S2, Supporting Information). The formation of hierarchical pores is beneficial for electrolyte infiltration and helps alleviate electrode volume expansion during cycles.^[^ ^21^ ^]^

Transmission electron microscopy (TEM) images of In_5_‐SPAN (Figure 1b–e; Figure S3a—c, Supporting Information) reveal no crystalline particles or lattice fringes within the nanofibers; instead, selected area electron diffraction (SAED) patterns show diffuse, continuous, and thick rings, confirming the amorphous nature of the In_5_‐SPAN composite fibers. The energy dispersive X‐ray spectroscopy (EDS) elemental mapping (Figure 1f; Figure S3d, Supporting Information) demonstrates a homogeneous distribution of C, N, S, and In throughout the fibers, while no observable indium aggregates are observed. X‐ray diffraction (XRD) analysis provides further evidence of the amorphous structure (Figure 1g; Figure S4, Supporting Information). The characteristic crystalline peaks of elemental sulfur and PAN disappear after sulfurization, replaced by a broad diffraction peak between 2θ≈20°and 30°, corresponding to the graphitic (002) plane of SPAN.^[^ ^22^ ^]^ Crucially, no diffraction peaks for crystalline indium sulfides are detected in the In_5_‐SPAN, consistent with the absence of crystalline domains in the TEM images (Figure 1b–f; Figure S3, Supporting Information). For reference, direct sulfurization of In(CH_3_COO)_3_ powder under an identical heating process yields crystalline In_2_S_3_ (JCPDS Card No.: PDF#73‐1366), as shown in Figure S5 (Supporting Information). These findings indicate that the indium is incorporated as amorphous In—S complexes uniformly distributed within the fibers. We infer that the uniform In–S bonding network and the covalent In─S─S linkages in the fibers effectively suppress indium sulfide crystallization and particle aggregation, resulting in an amorphous composite structure.

Thermogravimetric analysis (TGA) in Figure 1h qualitatively indicates an increase in sulfur content after indium incorporation into the SPAN composite.^[^ ^23^ ^]^ Inductively coupled plasma optical emission spectrometry (ICP‐OES) and elemental analysis (EA) are further performed to quantify the sulfur and active material fraction. The In_5_‐SPAN composite achieves a high active material (SPAN and In—S composite) loading of 47.4 wt.% with only 1.18 wt.% indium (Figure 1i; Table S1, Supporting Information). This represents an increase of 8.9 wt.% (≈23% relative) in active materials compared to SPAN powder prepared under identical conditions without indium. This significant increment can be attributed to the formation of In─S bonds (as will be elaborated in later sections), which stabilize additional sulfur in the composite, thereby raising the sulfur content beyond the normal limit for SPAN.

**Figure 2:**
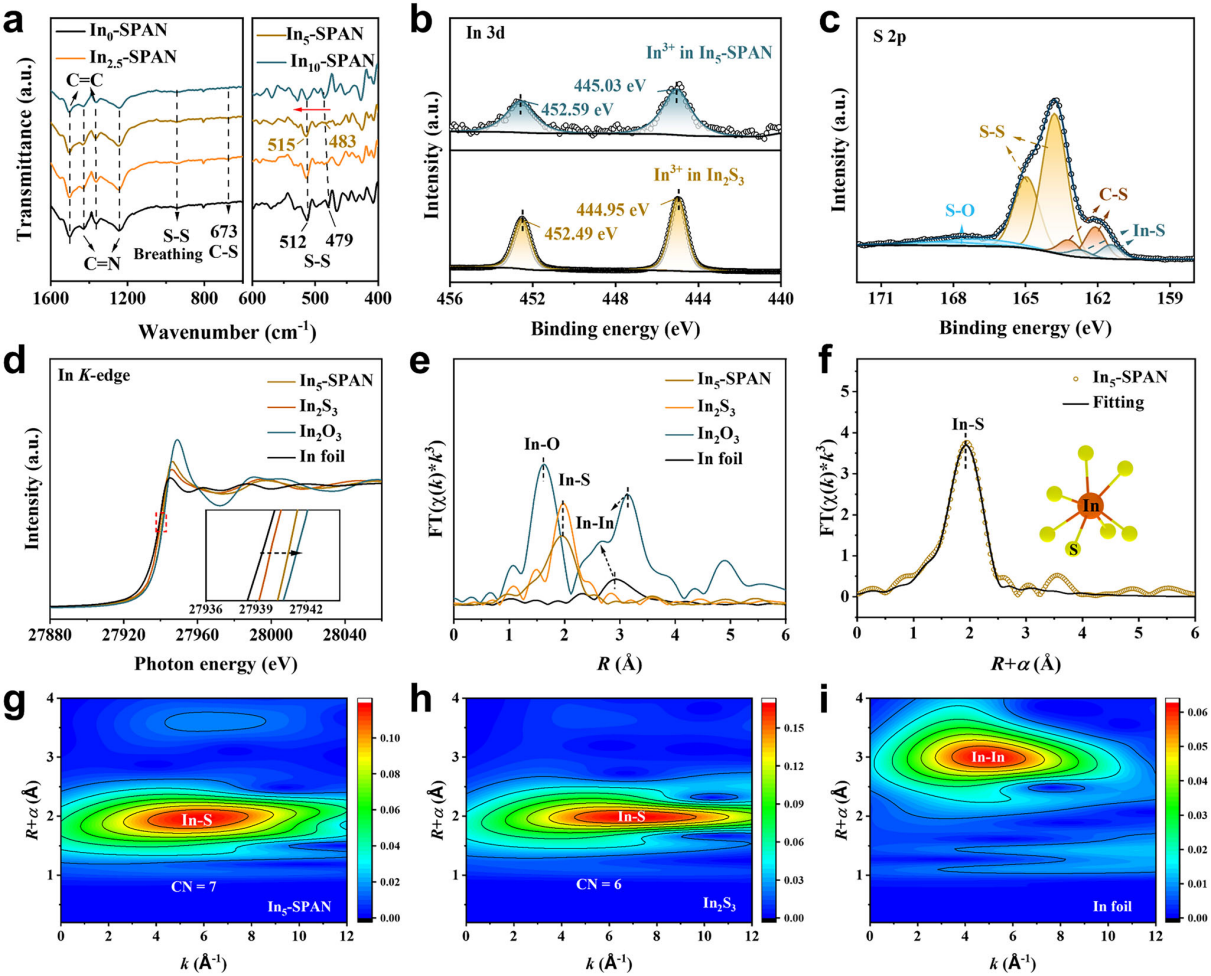
Molecular structure characterization of In_x_‐SPAN composites. a) FTIR spectra of In_x_‐SPAN. b) In 3d XPS spectra comparing In_5_‐SPAN with In_2_S_3_. c) S 2p XPS spectrum of In_5_‐SPAN. d) In *K*‐edge XANES spectra and e) Fourier transform of the k^3^‐weighted EXAFS (FT‐EXAFS) profiles of In_5_‐SPAN, In_2_S_3_, In_2_O_3_, and In foil. f) FT‐EXAFS spectra of In_5_‐SPAN (Inset shows the schematic model of the fitting results). g–i) Wavelet transform‐EXAFS (WT‐EXAFS) contour plots of In_5_‐SPAN, In_2_S_3_, and In foil.

The chemical structure of In_x_‐SPAN is analyzed using Fourier transform infrared (FTIR) spectroscopy and Raman analysis. FTIR spectra (**Figure** **2**a) of pristine SPAN show characteristic S─S stretching bonds at 512 and 479 cm^−1^. Upon indium incorporation, these S─S bonds shift to higher wavenumbers, indicating a lengthening of the sulfur chains due to strengthened In–S interactions.^[^ ^24^ ^]^ In contrast, the vibrational bonds associated with the SPAN polymer backbone, such as the C─S stretching at ≈673 cm^−1^ and the conjugated C ═ N/C ═ C vibrations, remain essentially unchanged, confirming that the PAN‐derived framework is intact despite In doping. Complementary Raman spectra (Figure S6 and Table S4, Supporting Information) display preserved SPAN signatures, including C─S (174, 307, and 367 cm^−1^), S─S (470 and 928 cm^−1^),^[^ ^25^ ^]^ and the carbon D and G bonds (1315, and 1544 cm^−1^). Notably, the D/G intensity ratio (I_D_/I_G_) slightly increases from 1.024 (In_0_‐SPAN) to 1.082 (In_10_‐SPAN), suggesting that indium incorporation introduces more disorder into the carbon matrix.

X‐ray photoelectron spectroscopy (XPS) spectra confirm the formation of indium‐sulfur bonds in In_5_‐SPAN. Survey XPS spectra (Figure S7, Supporting Information) show the expected C, N, S, and In signals. In the high‐resolution In 3d spectrum of In_5_‐SPAN (Figure 2b), we observe In 3d_5/2_ and In 3d_3/2_ double peaks at 445.03 and 452.59 eV, respectively. These binding energies are slightly higher than those of crystalline In_2_S_3_ (444.95 eV/452.49 eV),^[^ ^26^ ^]^ indicating that indium in the composite is bonded to sulfur and in a higher valence state than In^3+^ in In_2_S_3_, owing to the decreased electron density around the In nucleus.^[^ ^27^ ^]^ The S 2p spectrum of In_5_‐SPAN (Figure 2c) can be deconvoluted into four components^[^ ^22^ ^]^: In–S (161.45/162.75 eV), C–S (162.1/163.2 eV), S–S (163.8 eV/164.5 eV), and S–O (167.0/168.3 eV). The In–S component in In_5_‐SPAN aligns closely with that in an In_2_S_3_ reference (S 2p: 161.50/162.71 eV, Figure S8, Supporting Information), further confirming that indium is indeed bonded to sulfur in the composite. Based on these results, we conclude that after sulfurization, the indium is present as an amorphous In–S complex within SPAN, in which indium atoms exhibit a slightly higher effective valence than in crystalline In_2_S_3_, possibly due to the different local bonding environment.

To gain deeper insight into the indium coordination environment, we perform indium *K*‐edge X‐ray absorption fine structure (XAFS) analysis on In_5_‐SPAN, with In metal, In_2_S_3_, and In_2_O_3_ as references. The X‐ray absorption near‐edge structure (XANES, Figure 2d) of In_5_‐SPAN shows a distinct edge shift to higher energy compared to metallic In and In_2_S_3_, consistent with indium in In_5_‐SPAN being in a more oxidized state (in agreement with XPS results). The extended X‐ray absorption fine structure (FT‐EXAFS) spectra and their Fourier transforms in R‐space (Figure 2e,f; Figure S10c, Supporting Information) provide real‐space information on bond distances. In_5_‐SPAN exhibits a primary peak at ≈1.95 Å in the Fourier‐transformed EXAFS, which we assign to In—S bonds. This distance is slightly shorter than the In—S bond in crystalline In_2_S_3_ (1.98 Å) but significantly longer than the In‒O bond in In_2_O_3_ (1.62 Å),^[^ ^28^ ^]^ confirming a moderately contracted but still sulfur‐coordinated environment around In in the amorphous phase. Wavelet transform (WT)‐EXAFS analysis (Figure 2g–i; Figure S9, Supporting Information) further shows that In_5_‐SPAN and In_2_S_3_ have similar WT‐maximum, both at *k*≈6 Å^−1^ and *R* ≈1.9 Å, suggesting a similar type of In—S coordination in both, although less ordered in the amorphous case. Quantitative EXAFS fitting (Table S5 and Figure S10, Supporting Information) reveals an average coordination number of ≈7 in In_5_‐SPAN, which can be modeled as two subshells of ≈4 and 3 neighboring atoms at In—S distances of 2.46 ± 0.012 Å and 2.60 ± 0.017 Å, respectively. A plausible structural model derived from the fitting is illustrated in the insert of Figure 2f. By contrast, crystalline In_2_S_3_ has a lower average In coordination number of 6. The higher coordination number in amorphous In_5_‐SPAN indicates that indium atoms are surrounded by more sulfur atoms on average, which is characteristic of an amorphous network. This phenomenon is analogous to reports on other amorphous metal sulfides (e.g., TiS_4_ materials),^[^ ^15b^ ^]^ where disordered structure allows higher coordination numbers than the crystalline analog. These findings suggest that the indium precursor decomposes during sulfurization and transforms into amorphous In–S composites that interlink with the SPAN matrix and residual free sulfur. This integration into the polymer network and bonding with sulfur atoms effectively increases the amount of sulfur that can be stabilized in the cathode, as evidenced by the higher sulfur content in In_5_‐SPAN.

**Figure 3:**
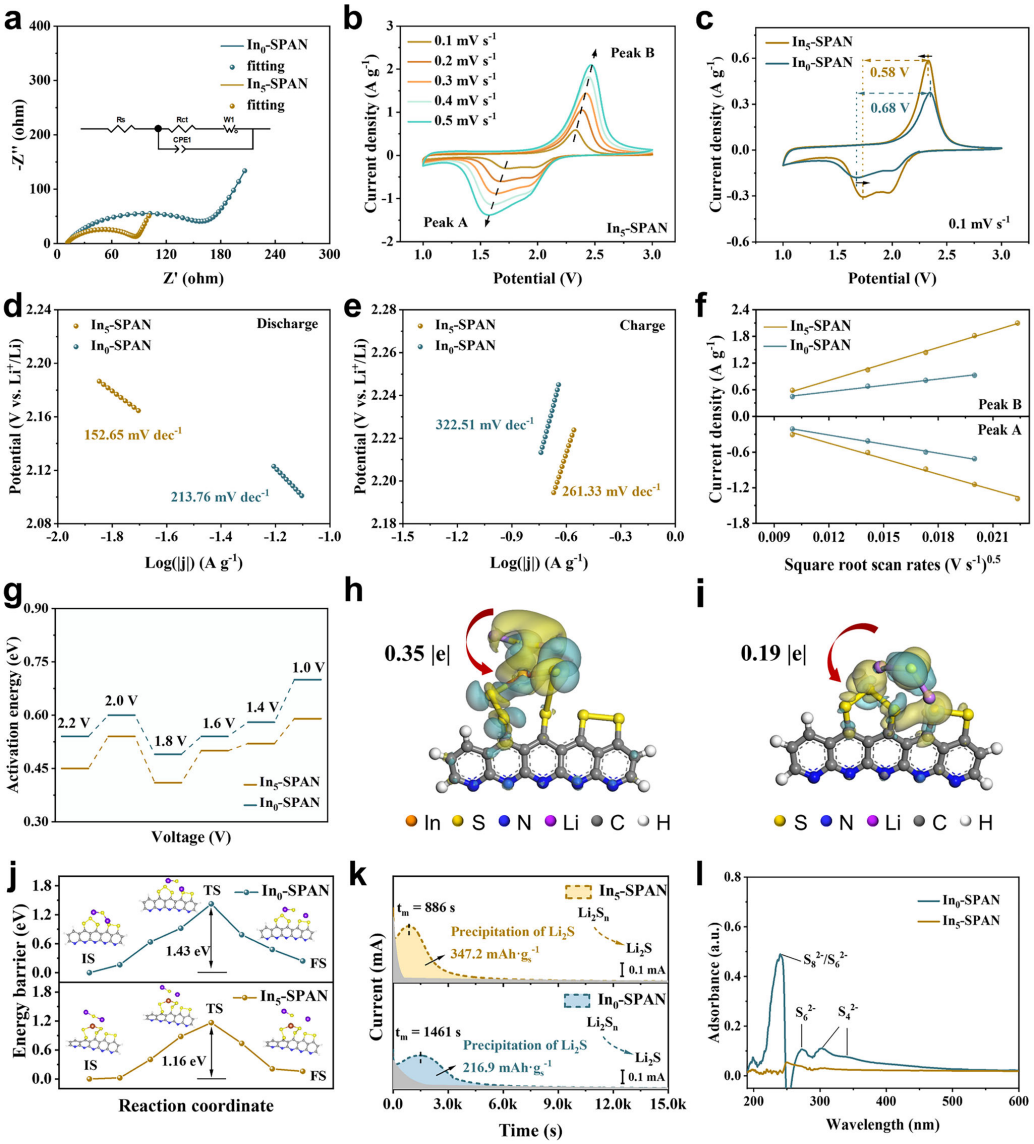
The kinetics evaluation of redox reactions in In_x_‐SPAN composites. a) EIS Nyquist plots with equivalent circuit model (inset). b) Cyclic voltammetry (CV) profiles of In_5_‐SPAN at scan rates spanning 0.1–0.5 mV s^−1^. c) CV profiles comparison of In_0_‐SPAN and In_5_‐SPAN at 0.1 mV s^−1^. d‐e) Corresponding Tafel plots at d) discharge and e) charge processes. f) Peak currents versus square root of scan rates of In_0_‐SPAN and In_5_‐SPAN. g) Activation energy evolution across voltages within the second discharge cycle. Bader charge density difference for h) In_5_‐SPAN and i) In_0_‐SPAN with Li_2_S. j) Decomposition energy barriers of Li_2_S. k) Potentiostatic discharge profiles of Li_2_S nucleation. l) UV–vis spectra of washing solutions collected from cycled In_0_‐SPAN and In_5_‐SPAN cathodes.

To evaluate the kinetic advantages of indium incorporation, we compare the electrochemical behavior of In_5_‐SPAN and undoped SPAN (In_0_‐SPAN) via impedance and voltammetry analyses. Electrochemical impedance spectroscopy (EIS) of fresh cells reveals that the charge transfer resistance (*R*_ct_) of In_5_‐SPAN is dramatically lower than that of In_0_‐SPAN (74 Ω vs 161 Ω, respectively), indicating much improved interfacial electron‐transfer kinetics with In in the cathode (**Figure** **3**a). Cyclic voltammetry (CV) at various scan rates further highlights the enhanced kinetics. The CV profiles of In_5_‐SPAN (Figure 3b; Figure S11, Supporting Information) exhibit substantially higher peak currents than those of In_0_‐SPAN, across scan rates from 0.1 to 0.5 mV s^−1^. In particular, at a slow scan of 0.1 mV s^−1^, In_5_‐SPAN shows a sharper and more pronounced reduction peak, whereas In_0_‐SPAN's peaks are smaller and more polarized. At this rate, the reduction peak of In_5_‐SPAN occurs at a slightly higher voltage and the oxidation peak at a slightly lower voltage compared to In_0_‐SPAN (Figure 3c), resulting in a smaller overall peak separation (0.58 V for In_5_‐SPAN and 0.68 V for In_0_‐SPAN). The reduced peak‐to‐peak polarization confirms that In doping accelerates the sulfur redox reaction and lowers the kinetic barriers.^[^ ^29^ ^]^ Additionally, a CV at an even slower scan (0.05 mV s^−1^, Figure S12, Supporting Information) for In_5_‐SPAN reveals two cathodic peaks at 2.07 and 1.77 V, respectively, corresponding to the rapid reduction of covalently bound sulfur and subsequent formation of insoluble Li_2_S_2_/Li_2_S. In contrast, a single anodic peak at 2.28 V is observed, which is associated with the reversible regeneration of SPAN.^[^ ^6b^ ^]^ This behavior indicates that indium‐modified SPAN follows a closed‐loop solid‐phase conversion mechanism without forming long‐chain polysulfides.

Furthermore, Tafel analysis is conducted to quantify the reaction kinetics. As shown in Figure 3d,e, the In_5_‐SPAN electrode exhibits a significantly lower Tafel slope than In_0_‐SPAN in both discharge and charge. Specifically, during discharge, the slope for In_5_‐SPAN is 152.65 mV dec^−1^, substantially lower than the 213.76 mV dec^−1^ of In_0_‐SPAN. Likewise, during charge, the slope for In_5_‐SPAN is 261.33 mV dec^−1^ versus 322.51 mV dec^−1^ for In_0_‐SPAN. The smaller Tafel slopes indicate faster reaction kinetics for the conversion between covalently bonded sulfur and Li_2_S_2_/Li_2_S in the indium‐modified cathode. Additionally, the Li‐ion diffusion kinetics are evaluated using Randles–Sevick analysis of the CV data.^[^ ^6b^ ^]^ Figure 3f shows the linear fits of peak current (*I*_p_) versus the square root of scan rate (*v* ^0.5^) for the redox peaks. According to the Randles–Sevcik equation, the Li^+^ diffusion coefficients (*D*_Li_^+^) for In_5_‐SPAN at the oxidation and reduction peaks are 2.13 × 10^−7^ cm^2^·s^−1^ and 1.14 × 10^−7^ cm^2^·s^−1^, respectively (Figure S13, Supporting Information). These values are ≈4.6 times and 2.1 times higher, respectively, than those for In_0_‐SPAN (4.6 × 10^−8^ cm^2^·s^−1^ and 5.4 × 10^−8^ cm^2^·s^−1^). The significantly increased Li^+^ diffusion coefficients indicate that the amorphous In–S phase effectively reduces the solid‐phase diffusion barrier, promoting faster Li⁺ transport through the SPAN matrix. Furthermore, Tafel analysis is conducted to quantify the reaction kinetics.

To elucidate the intrinsic mechanism of the kinetic enhancement, we carry out temperature‐dependent EIS measurements and density functional theory (DFT) simulations. Impedance spectra are collected at multiple temperatures (293–323 K) for cells at specific voltages from 2.2 V down to 1.0 V in the second discharge (Figure S14, Supporting Information), and the Arrhenius behavior of *R*_ct_ is analyzed (Figures S15 and S16, Supporting Information). From the slope of ln(1/*R*_ct_) versus 1/*T* Arrhenius equation (Figure S17, Supporting Information),^[^ ^31^ ^]^ we extracted the charge‐transfer activation energy (*E*_a_) at each discharge voltage. As seen in Figure 3g, both In_5_‐SPAN and In_0_‐SPAN exhibit similar trends during discharge, but In_5_‐SPAN has a consistently lower *E*_a_ value across all stages. This systematic reduction in activation energy strongly corroborates the enhanced redox kinetics imparted by the amorphous In–S composites. Based on the XAFS analysis in Figure 2f and previous reports on heteratom‐doped SPAN,^[^ ^6^, ^7^, ^13^ ^]^ a simplified structural model of In_5_‐SPAN is proposed. Geometry optimization results (Figure S18, Supporting Information) indicate that indium sites form strong interactions with sulfur atoms, thereby stabilizing them in the polymer matrix. The oxidation kinetics of Li_2_S are widely regarded as a key rate‐limiting step owing to its intrinsically low electron conductivity.^[^ ^31^ ^]^ Bader charge analysis of Li_2_S adsorbed on In_5_‐SPAN and In_0_‐SPAN is conducted to examine charge redistribution and net charge transfer upon adsorption (Figure 3h,i). Yellow zones in the charge density difference maps indicate electron accumulation, while green zones suggest electron depletion. Notably, the net charge transfer from S of Li_2_S to the substrate matrix is higher for In_5_‐SPAN‐Li_2_S (0.35 |e|) than that of In_0_‐SPAN‐Li_2_S (0.19 |e|), indicating enhanced electronic coupling and chemisorption abilities. In addition, reaction pathway calculations (Figure 3j) show that the energy barrier for Li_2_S dissociation is 1.16 eV on In_5_‐SPAN, which is significantly lower than the 1.43 eV observed on In_0_‐SPAN, suggesting that the In—S network promotes Li─S bond cleavage and accelerates reaction kinetics during battery charging. According to the nucleation and dissolution measurements of Li_2_S (Figure 3k; Figure S19, Supporting Information), the Li_2_S deposition profile demonstrate an earlier current peak of 886 s and a higher capacity of 347.2 mAh g^−1^ for In_5_‐SPAN than those of In_0_‐SPAN (1461 s, 216.9 mAh g^−1^), indicating elevated reduction kinetics from lithium polysulfides (LiPSs) to Li_2_S. Furthermore, the In_5_‐SPAN shows a higher dissociation capacity (810.4 mAh g^−1^) than In_0_‐SPAN (647.4 mAh g^−1^), indicating a lower dissociation barrier of Li_2_S for In_5_‐SPAN. Additionally, the Li_2_S dissociation performance is further revealed by LSV curves and calculated Tafel slopes accordingly (Figure S20, Supporting Information). The In_5_‐SPAN electrode exhibits a lower onset potential (−0.46 V) and a smaller Tafel slope (238.3 mV dec^−1^) relative to those for In_0_‐SPAN (−0.42 V, 331.3 mV dec^−1^), along with a higher current response, suggesting a faster reaction kinetics of Li_2_S oxidation. These experiments and theoretical calculations demonstrate that the formation of In—S coordination can facilitate the bidirectional conversion between SPAN and Li_2_S, thereby enhancing the redox kinetics.

The adsorption energies of LiPSs have been calculated in order to provide a theoretical evaluation of the anchoring capability of the In—S network toward sulfur species. The configurations of LiPSs adsorbed on samples are optimised and presented in Figures S21 and S22 (Supporting Information). As demonstrated in Figure S23 (Supporting Information), the adsorption energies for LiPSs on In_5_‐SPAN are higher than those on In_0_‐SPAN, indicating a much stronger anchoring effect induced by the incorporation of In—S network, which effectively suppresses the dissolution and diffusion of LiPSs, while enabling their re‐anchoring through reconfigurable In—S coordination within the SPAN matrix. Furthermore, we probe the effect of indium on polysulfide dissolution by UV–vis spectroscopy of electrolytes after cycling. Figure 3l shows the UV–vis absorption of the DOL/DME solvent after thoroughly rinsing the cycled cathodes. The electrolyte from an In_0_‐SPAN cathode displays distinct adsorption peaks at ≈350 and ≈300 nm, corresponding to S_4_^2−^ species, and additional peaks ≈280 and 250 nm are associated with S_6_^2−^ and S_8_^2−^/S_6_^2−^ species. S_n_^2−^ (4 ≤ n ≤ 8) species are presumably generated from the disproportionation reaction of low‐order intermediates S_n_^2−^ (n ≤ 4).^[^ ^6^, ^7^ ^]^ The above results indicate that the dissolution and diffusion of polysulfides from the In_0_‐SPAN cathode emerge, resulting in steady capacity decay. In contrast, polysulfide dissolution is significantly inhibited for In_5_‐SPAN owing to the fast redox conversions of intermediate polysulfides and the binding sites provided by indium that immobilize polysulfides, preventing their diffusion. This suppression of polysulfide shuttling contributes to the superior long‐term stability of In_5_‐SPAN, as discussed next.

**Figure 4:**
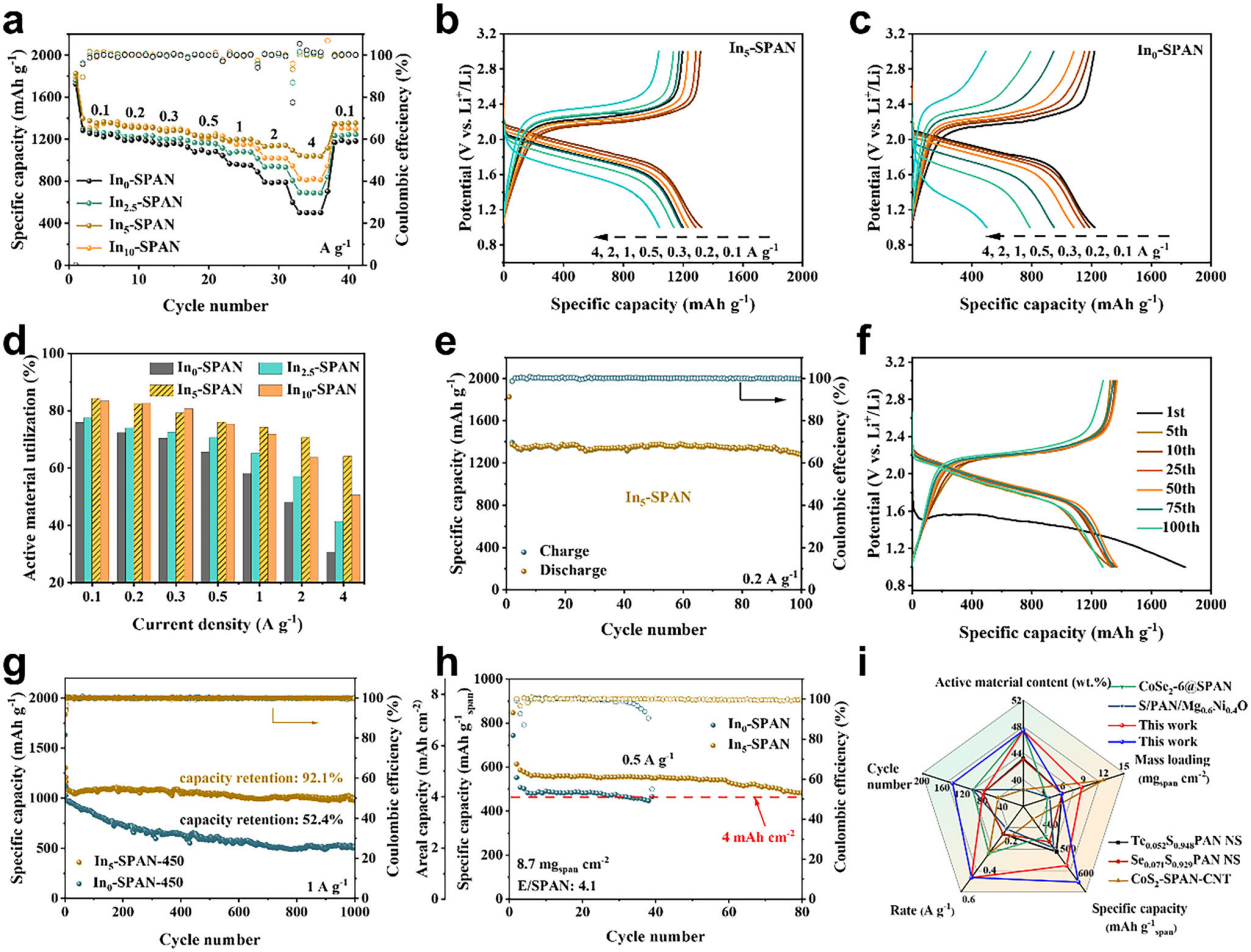
Electrochemical performance of In_x_‐SPAN‐based batteries. a) Rate properties of In_x_‐SPAN. b) Voltage profiles of In_5_‐SPAN and c) In_0_‐SPAN at current densities spanning 0.1–4 A g^−1^. d) Active material utilization efficiency of In_x_‐SPAN across tested current densities. e) Cycle property of In_5_‐SPAN at 0.2 A g^−1^ and f) corresponding voltage evolution during cycling. g) Long‐cycle life of the In5‐SPAN and In0‐SPAN cathodes at 1 A g^−1^. h) Cycling property at 0.5 A g^−1^ under high areal mass loading and lean electrolyte conditions. i) Radar chart benchmarking key performance metrics against prior studies.

The rate capability of the In_x_‐SPAN is investigated at current rates ranging from 0.1 to 4 A g^−1^. As shown in **Figure** **4**a,b, upon cycling, the In_5_‐SPAN cathode delivers the highest reversible specific capacities of 1355, 1307, 1281, 1225, 1199, 1141, and 1035 mAh·g^−1^ at current densities of 0.1, 0.2, 0.3, 0.5, 1, 2, and 4 A g^−1^, respectively. Notably, when the current is switched back to 0.1 A g^−1^, the specific capacity recovers to 1351 mAh·g^−1^, demonstrating the excellent reversibility and stability of the electrode. By contrast, for the In_0_‐SPAN cathode, the specific capacity drops more steeply with increasing rates, 1244, 1183, 1151, 1081, 950, 787, and 500 mAh·g^−1^ when cycled from 0.1 to 4 A g^−1^ (Figure 4c). These capacity increments, especially at 4 A g^−1^ are attributed to the bidirectional acceleration of solid‐solid conversions enabled by the reconfigurable In—S coordination. The In_5_‐SPAN cathode achieves over 85% active material utilization at 0.1 A g^−1^ and 76.4% capacity retention at 4 A g^−1^, whereas In_0_‐SPAN utilizes only 76% of active material and retains only 40% capacity (Figure 4d). The higher utilization and superior rate tolerance explicitly validate the kinetic acceleration role of In‐modification. These results illustrate the dramatic improvement in rate performance imparted by indium incorporation, consistent with the faster kinetics discussed earlier. Furthermore, galvanostatic charge/discharge (GCD) profiles at 0.2 A g^−1^ and 1 A g^−1^ reveal a 25% reduction in polarization potential for In_5_‐SPAN (0.32 and 0.46 V, respectively) relative to In_0_‐SPAN (0.38 and 0.61 V, in Figure S24, Supporting Information), aligning with the CV observations (Figure 3c). This underscores the role of indium in mitigating electrochemical irreversibility and accelerating redox kinetics.

We assess the long‐term cycling performance under various conditions. Under moderate sulfur loading (1.5 mg cm^−2^) and a current density of 0.5 A g^−1^, the In_5_‐SPAN cathode exhibits remarkable stability, retaining nearly 100% of its initial capacity after 100 cycles. In_10_‐SPAN and In_2.5_‐SPAN also show excellent retention but slightly lower than In_5_‐SPAN, while In_0_‐SPAN fades more noticeably (Figure S25, Supporting Information). Extended cycling at 0.2 A g^−1^ confirms this superiority, where In_5_‐SPAN demonstrates a steady high capacity of ≈1351 mAh·g^−1^ for over 100 cycles with ≈100% coulombic efficiency (Figure 4e,f). Even at a high current of 1A g^−1^, In_5_‐SPAN maintains 972 mAh·g^−1^ (670 Wh Kg^−1^_cathode_) after 300 cycles, starting from 1140 mAh·g^−1^ (790 Wh Kg^−1^_cathode_) at the first cycle, corresponding to a capacity decay rate of only 0.049% per cycle. In stark contrast, the In_0_‐SPAN decays rapidly down to 500 mAh·g^−1^ (318 Wh Kg^−1^_cathode_) by 175 cycles (Figure S26a,b, Supporting Information). The dramatically improved capacity retention and cycling stability of In_5_‐SPAN further confirms that the indium‐sulfur coordination strategy accelerates kinetics and effectively inhibits the deleterious polysulfide shuttle and related degradation processes.

To verify the broad applicability of indium modification, we also prepare SPAN fibers sulfurized at a higher temperature of 450 °C (denoted In_5_‐SPAN‐450) and test them in long‐term cycling. Impressively, In_5_‐SPAN‐450 achieves 92.1% capacity retention over 1000 cycles at 1 A g^−1^, while the control In_0_‐SPAN‐450 retains only 52.4% under the same conditions (Figure S4c, Supporting Information; Figure 4g). Crucially, the In_5_‐SPAN cells also show excellent properties under practical high‐loading, lean‐electrolyte conditions. We assembled cells with a high areal mass loading of 8.7 mg cm^−2^ and a low electrolyte‐to‐SPAN mass ratio (E/SPAN) of 4.1 µL mg^−1^. Under these stringent conditions, the In_5_‐SPAN delivers a high areal capacity of ≈4.8 mAh cm^−2^ initially and maintains a specific capacity of 1048 mAh·g^−1^_sulfur_ (484 mAh g^−1^ based on total SPAN composite mass) after 80 cycles at 0.5 A g^−1^. By contrast, the In_0_‐SPAN cathode fails to sustain high capacity under these conditions, dropping to ≈400 mAh g^−1^_sulfur_ by 80 cycles, as seen in Figure 4h. We summarize the preparation methods and key performance metric of our In_5_‐SPAN cathode relative to other reported heteroatom‐doped SPAN cathodes (e.g., Te, Se, Co, and Ni) and recently reported Li‒S batteries in Tables S7 and S8 (Supporting Information). The radar chart in Figure 4i illustrates that our sample achieves a unique combination of high active material content and excellent cyclic performance.

**Figure 5:**
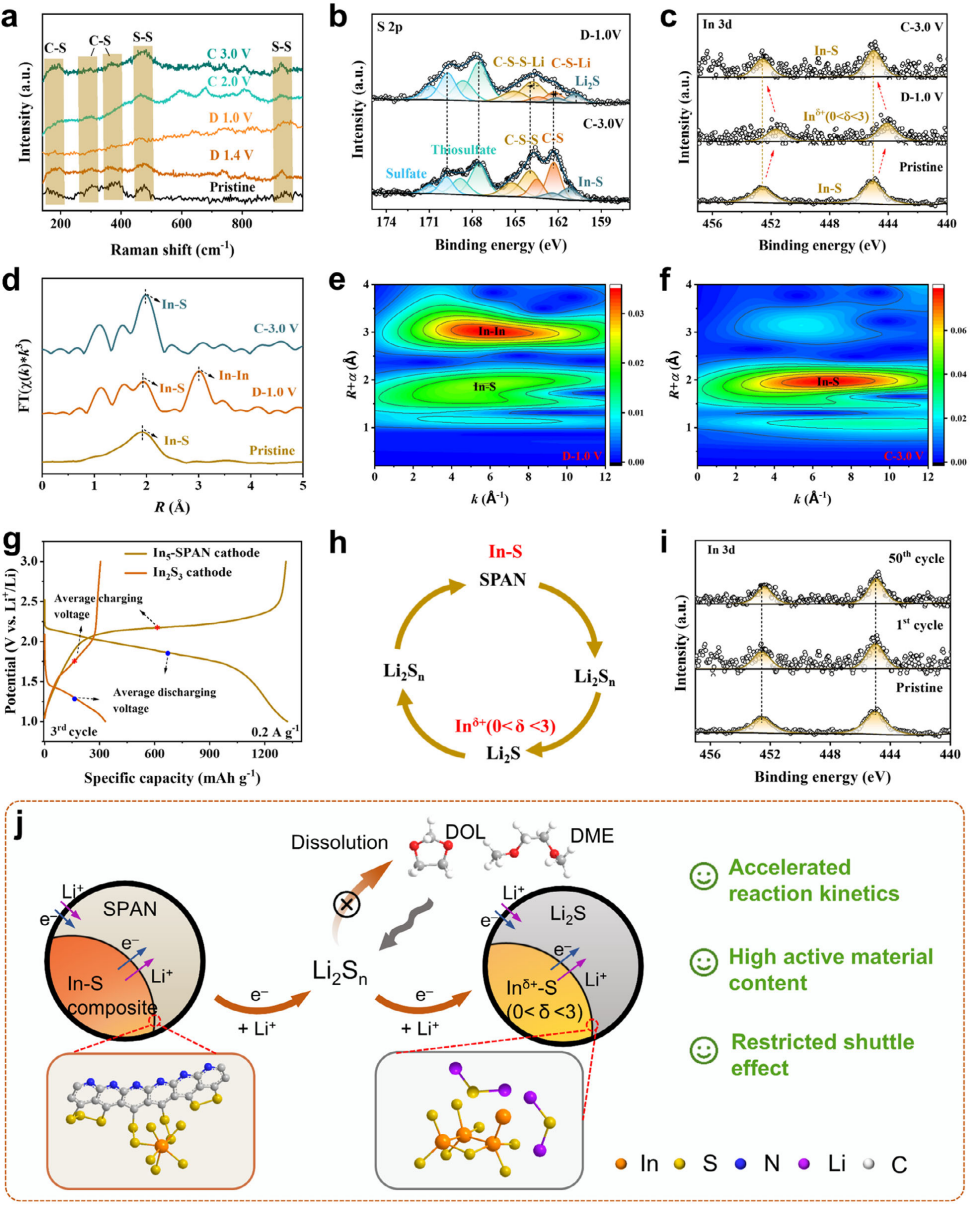
The structural and chemical evolution characterization of In_5_‐SPAN electrodes. a) Ex situ Raman spectra of In_5_‐SPAN electrodes at varying voltage during the first cycle. b,c) XPS spectra of b) S 2p and c) In 3d for the In_5_‐SPAN electrodes in fully discharged and fully charged states. d) k^3^‐weighted FT‐EXAFS profiles of In_5_‐SPAN electrodes at pristine, fully discharged, and fully charged states. e,f) WT‐EXAFS contour plots of In_5_‐SPAN at e) fully discharged and f) fully charged states. g) Galvanostatic charge/discharge profiles of In_5_‐SPAN and In_2_S_3_ cathode at 0.2 A g^−1^. h). The SPAN redox reaction cycle in the presence of the In–S composite catalyst. i) In 3d XPS spectra of In_5_‐SPAN electrodes at pristine, 1st cycle, and 50th cycle. j) Schematic diagram of the proposed reaction process within the reconfigurable In—S coordination system.

To reveal the structural and chemical evolution of the indium–sulfur sites during battery operation, we conduct ex situ Raman, XPS, and XAFS measurements on In_5_‐SPAN electrodes at various states of charge (**Figure** **5**). As shown in Figure 5a, the intensities of the C‒S bond and S‒S bond markedly decrease upon full discharge, then recover upon charging, suggesting high reversibility. To examine the chemical states in more detail, we turn to high‐resolution XPS. In the S 2p spectrum of the fully discharged state, the original peaks for the C─S─S_x_ (S 2p_3/2_: 164.0, S 2p_1/2_: 165.3 eV) and C─S (S 2p_3/2_: 162.3, S 2p_3/2_: 163.5 eV) bonds have shifted to lower binding energies (C─S─S‒Li: 163.8 eV/165.1 eV; C‒S‒Li: 162.2 eV/163.4 eV; Li_2_S: 160.7 eV/162.1 eV), due to the formation of the Li─S bond after lithiation (Figure 5b).^[^ ^3^, ^33^ ^]^ Upon charging to 3.0 V, the C─S─S_x_ and C─S bonds are reversibly restored, while Li_2_S transforms into an In—S bond (S 2p_3/2_: 161.4 eV, S 2p_1/2_: 162.7 eV), aligning with the pristine spectra in Figure 2c. Concurrently, the doublet peaks of In 3d shift from In^3+^ (In—S, 445.03 eV/452.59 eV) to In^δ+^ (0<δ<3, 444.11 eV/451.63 eV) during discharge,^[^ ^34^ ^]^ reversibly returning to In^3+^ (445.01 eV/452.57 eV) after charge to 3.0 V(Figure 5c).^[^ ^17^, ^35^ ^]^ This dynamic In–S ↔ In^δ+^ interconversion demonstrates a sulfur‐coordination reconfiguration mechanism stabilizing polysulfide intermediates. Furthermore, FT‐EXAFS spectra (Figure 5d) further demonstrate the reversible transformation of In─S bond (1.95 Å) and In─In bond (3 Å) during cycling. Meanwhile, WT‐EXAFS contour plots exhibit a prominent peak at R≈3 Å (In─In bond region) and a minor peak at R≈1.90 Å (In─S bond region) after discharge to 1.0 V (Figure 5e). Upon charging to 3.0 V, the In─In bond peak intensity diminishes significantly, while the In─S bond peak intensity increases and shifts back to R≈1.95 Å (In─S bond region) (Figure 5f), further confirming the high reversibility of In─S bonds within In_5_‐SPAN. Notably, the dynamic breakage and formation of metal‒sulfur bonds are also reported by others.^[^ ^36^ ^]^ Quantitative EXAFS fitting reveals that at 1.0 V (fully discharged), the indium has an average of ≈3.4 In neighbors and ≈2.5 S neighbors, implying significant In─S bond cleavage and clustering of indium, which provide abundant exposed active sites that adsorb and confine the dissolved polysulfide, mitigating their diffusion and shuttle effect. After charging to 3.0 V, the average In–S coordination number recovers to 5.1 (5.1±0.6), consistent with the reformation of In─S bonds (Table S6, Supporting Information).

To further unveil the dynamic cleavage/formation of In─S bonds and their redox potentials, crystalline In_2_S_3_ is prepared and assembled as Li||In_2_S_3_ batteries. As shown in Figure S27a (Supporting Information), the dQ/dV curves of In_2_S_3_ demonstrate distinct lithiation plateaus at 1.39 and 1.16 V, corresponding to Li⁺ intercalation and In—S bond cleavage, respectively. This observation is also supported by the XRD characteristic peaks and the TEM lattice fringes of In metal (Figure S28, Supporting Information). Upon charging, the characteristic peak of In_2_S_3_ at 27.4° reappears, confirming the feasibility of dynamic cleavage/formation for In—S bonds. According to the discharge‐charge curves (Figure 5g) and dQ/dV profiles of In_5_‐SPAN and In_2_S_3_ (Figure S27, Supporting Information). The breakage of In—S bonds typically occurs below 1.6 V, while the average discharge voltage of In_5_‐SPAN is 1.85 V. This delayed conversion process not only enhances the ability of metal sulfides to accelerate SPAN‐to‐Li_2_S conversion but also generates active sites that adsorb dissolved polysulfides, thereby inhibiting their dissolution. Upon charging, In—S bond reconstruction occurs below 2.1 V, which is lower than the charging plateau voltage of In_5_‐SPAN (2.17 V), thus facilitating sulfur matrix bonding in SPAN (Figure 5h). Consequently, the In_5_‐SPAN electrode exhibits significantly reduced polarization and improved redox kinetics. This dynamic modulation of valence bonds synchronously accelerates Li_2_S deposition and sulfur matrix reconstruction, endowing the electrode with higher sulfur utilization and cycling stability (Figure 5j).

Finally, we evaluated the stability of the indium‐sulfur bonding over many cycles. The In 3d XPS spectra of In_5_‐SPAN after 50 cycles (Figure 5i) remain virtually unchanged compared to the first cycle, indicating that the reversible In–S/In–In conversion is maintained and no new irreversible indium species accumulate. We did detect a small degree of indium dissolution into the electrolyte over long cycling, where inductively coupled plasma‐optical emission spectrometer (ICP‐OES) analysis of the electrolyte before cycling shows ≈9.8 µg mL^−1^ of In^3+^ leached from the cathode (Figure S29, Supporting Information), and XPS depth profiling of a cycled cathode shows a gradient of indium concentration (higher at the surface, Figure S30, Supporting Information). Notably, such partial indium dissolution has been reported in other indium‐based Li–S cathodes as well.^[^ ^3^, ^18^, ^37^ ^]^ To assess the influence of dissolved In on the anode side, we performed the Ar^+^ etched XPS analysis of the Li anode cycled with In_5_‐SPAN. As shown in Figure S31 (Supporting Information), the In 3d XPS spectrum displays two binding energies at 440.7 and 448.3 eV, corresponding to Li–In alloy, while the additional peaks at 442.4 and 450.0 eV are assigned to metallic In^0^.^[^ ^20^ ^]^ A Li 1s peak at ≈56.1 eV further confirms the presence of Li─In bonding.^[^ ^37^ ^]^ Surface and cross‐sectional SEM–EDS mappings also reveal the distribution of indium on the lithium surface. These results demonstrate indium migration and Li–In intermetallic layer formation during cycling, which stabilizes the interface and enhances cycling performance. Notably, in our cells with 8.7 mg cm^−2^ and an E/SPAN ratio of 4.1, after 30 cycles the lithium anode from the In_5_‐SPAN cell shows a smoother and more uniform surface in SEM images (Figure S32, Supporting Information) compared to the anode from an In_0_‐SPAN cell, suggesting that the indium‐containing species mitigate dendrite formation and help stabilize the anode. Overall, the indium in In_5_‐SPAN is mostly retained and remains active over prolonged cycling, with minor dissolution that fortuitously contributes to anode protection.

## 3 Conclusion

In summary, we have developed a porous SPAN fiber cathode embedded with an amorphous indium‐sulfur complex via electrospinning and sulfurization. The inclusion of indium markedly increases the active sulfur content of SPAN (reaching 47.4 wt.% in In_5_‐SPAN) owing to the higher sulfur coordination number (≈7) of indium in the amorphous phase and the reversible breaking/forming of In─S bonds. Both theoretical calculations and experiments indicate that the reconfigurable In—S coordination improves the lithium‐ion transport in the cathode and lowers the energy barrier of S─S bond conversion, facilitating rapid bi‐directional conversion between Li_2_S/Li_2_S_2_ and low‐order intermediate Li_2_S_n_ (n≤ 4) within SPAN. Consequently, a remarkable reversible capacity of 1035 mAh·g^−1^ is achieved at 4 A g^−1^, accompanied by capacity retention of 972 mAh·g^−1^ at 1 A g^−1^ after 300 cycles. Moreover, at a high loading of 8.7 mg cm^−2^ and lean electrolyte condition with E/SPAN = 4.1, an exceptional capacity of 1048 mAh·g^−1^ is still sustained after 80 cycles at 0.5 A g^−1^. This work demonstrates a new strategy that integrates a reversible metal–sulfur bond reconfiguration with structural electrode design, achieving simultaneous improvements in active material content and redox kinetics. Such an approach provides fresh insights for designing high‐performance Li‐S batteries and could be extended to other electrochemical systems where dynamic bonding can be leveraged for performance gains.

## 4 Experimental Section

### Chemicals and Materials

All chemicals are commercially available and without further purification after received. Polyacrylonitrile (Mw = 150 000) and Indium acetate were purchased from Sigma–Aldrich Co., Ltd., Shanghai, China. Polyethylene glycol (PEG) was bought from Y F Chemical Co., Ltd. Sulfur (S, ≥99.5 wt.%) was provided by Shanghai Chemical Corporation. Carboxymethyl cellulose (CMC) was provided by Dow Chemical Company, while styrene‐butadiene rubber (SBR) was bought from Shengzheng Kejing Star Technology CO., Ltd. N, N‐Dimethylformamide (DMF) was purchased from Sinopharm Chemical Reagent Co., Ltd., Shanghai, China. 1,3‐dioxolane (DOL), 1,2‐dimethoxyethane (DME) and lithium bis(trifluoromethanesulfonyl) imide (LiTFSI) were brought from Alfa Aesar.

### Synthesis of In_x_‐SPAN Fibers

The nanofibers were prepared via electrospinning. A certain amount of indium acetate powder (0.015, 0.03, 0.06 g) was added into 6 mL of DMF and then ball mixed with zirconium beads. After vigorous stirring for 2 h, PAN (0.6 g) and PEG (0.22 g) were dissolved to form a homogeneous solution. Then, the well‐mixed solution was fed into a syringe for electrospinning. The electrospinning process was operated at 14.0 kV with a pushing rate of 0.9 mL h^−1^, the distance between the collector and the spinneret was set to 15 cm, and the humidity was controlled between 30% and 35% to obtain uniform nanofibers.

### Synthesis of In_x_‐SPAN Electrodes

The as‐prepared fibers and the commercial sulfur powder (1:4, w/w) were mixed and covered with aluminum foil in a crucible. Then, the composites were heated at 380 °C for 6 h in an Ar atmosphere with a ramping rate of 5 °C min^−1^. The extra sulfur was removed by heating at 250 °C for 4 h in a flowing Ar atmosphere. The obtained composites were designated as In_x_‐SPAN, where x is the mass ratio of In_2_(CH_3_COO)_3_ and PAN (Table S1, Supporting Information). For comparison, the composites without PEG addition were also prepared under the same conditions.

### Materials Characterization

The morphologies and structures were characterized by scanning electron microscopy (SEM, ZEISS Gemini SEM300) and Transmission electron microscopy (TEM, JSM2100). The In *K*‐edge X‐ray adsorption fine spectroscopy (XAFS) was recorded at BL14W1 station in Shanghai Synchrotron Radiation Facility. The contents of C, H, N, and S in samples were detected by organic elemental analysis (EA, Elemental UNICUBE), while the In concentrations were conducted on the inductively coupled plasma optical emission spectroscopy (ICP‐OES, Agilent 7850). The compositions and chemical states of samples were characterized by X‐ray photoelectron spectroscopy (XPS) performed on Thermo Fisher ESCALab 250Xi system with Al/K anode (photon energy = 1486.6 eV) mono X‐ray source, and the sputtering source was Ar^+^ clusters. Thermal stability and active content were evaluated by a thermogravimetric (TG) analysis system (HITACHI STA200) with a heating rate of 10 °C min^−1^ from room temperature to 800 °C. The nitrogen sorption isotherms were collected by a Quantachrome Autosorb IQ system at a liquid‐nitrogen temperature. The powder X‐ray diffraction (XRD) patterns were measured by X‐ray diffractometer (DX‐2700BH) using Cu Kα as the radiation source. Fourier transform infrared (FTIR) spectra were collected with Bruker MPA Near IR. Raman spectra were recorded by Thermal Fisher‐Dxr 3xi with an excitation wavelength of 532 nm.

### Electrochemical Measurements

The In_x_‐SPAN composites were fully ground and then mixed with Super P, CMC, SBR with a mass ratio of 8:1:0.5:0.5 in deionized water to form a uniform slurry. After that, the slurry was coated on Al foil (18 µm) and dried at 60 °C overnight to remove the residual solvent. A circular disk of area 1.13 cm^2^ was punched with the areal mass loading of 1.5 mg cm^−2^. For high‐mass loading films, the electrode was prepared by coating the same slurry on carbon paper instead of Al foil. The as‐prepared In_x_‐SPAN electrodes were assembled as CR2032 coin cells with lithium anode, PP separator (celgard2325), and electrolyte in an Ar‐filled glove box. The ether electrolyte consisted of 1 m lithium bis(trifluoromethanesulfonyl)imide (LiTFSI) and 2% LiNO_3_ dissolved in 1,3‐dioxolane (DOL) and 1,2‐dimethoxyethane (DME) solution (1:1 v/v). Only 40 µL of electrolyte was added to each cell unless specified. Galvanostatic charge/discharge (GCD) was conducted with a LAND CT2001A system (Wuhan, China) within a voltage window of 1.0–3.0 V (vs Li/Li^+^). Cyclic voltammetry (CV) was measured on an electrochemical workstation (PGSTAT302N, metrohm, Switzerland) with scan rates of 0.1–0.5 mV s^−1^.

Electrochemical impedance spectroscopy (EIS) was recorded in the frequency range of 0.01–10 000 Hz with an amplitude of 5 mV on the CHI660E electrochemical workstation. For a temperature‐dependent EIS test, the temperature was maintained in an oven at 283, 293, 303, and 313 K, respectively. If not specified, the specific capacities and current densities of cells were calculated based on the mass of sulfur in In_x_‐SPAN.

### Measurements of Li_2_S Nucleation and Dissolution

Sublimed sulfur and Li_2_S with a weight ratio of 5:1 were mixed and added in the above ether electrolyte and then vigorously stirred at 60 °C for 24 h to obtain 0.2 m Li_2_S_6_ solution. For Li_2_S nucleation/dissociation experiments, 20 µL of 1 m LiTFSI and 20 µL of 0.2 m Li_2_S_6_ solution were utilized as the electrolyte to assemble the coin cells. The assembled batteries were performed on an electrochemical workstation (PGSTAT302N), and discharged galvanostatically at 0.112 mA to 2.09 V. After that, the precipitation of Li_2_S was conducted at 2.05 V under a potentiostatic discharge program until the current dropped below 10^−6^ A. Following the completion of the Li_2_S nucleation, the cells were subjected to charge with a constant potential of 2.4 V to conduct the Li_2_S dissolution measurement until the current dropped below 10^−6^ A. The Li_2_S nucleation capacity was calculated by the integral area of the curve via Faraday's Law.

### Linear‐Sweep Voltammetry (LSV) Measurements

LSV tests were conducted in a three‐electrode configuration to evaluate the catalytic activities and kinetics of various composites. The cells consisted of an In_5_‐SPAN cathode working electrode, a saturated Ag/AgCl reference electrode, and a platinum sheet counter electrode. After immersing in a 0.1 m Li_2_S/methanol electrolyte, the LSV measurements were carried out from −0.8 to 0.0 V at a scan rate of 5 mV s^−1^.

### Lithium‐Ion Diffusion Coefficient Evaluation

The coefficient of Li^+^ diffusion was estimated by the Randles–Sevick equation:

(1) $$I_{p}=\left(2.69\times 10^{5}\right)n^{1.5}AD_{Li^{+}}^{0.5}C_{Li^{+}}v^{0.5}$$

where *I*_p_ is the peak current based on the CV curves at varying scan rates, *n* represents the electron number transferred in the reaction (*n* = 2), A is the electrode area (*A* = 1.14 cm^2^), *D*_Li_^+^ represents the Li^+^ diffusion coefficient (cm^2^ s^−1^), *C*_Li_^+^ is the concentration of Li^+^ in electrolyte (*C*_Li_^+^ = 1 mol L^−1^) and *v* stands for the scanning rate (V s^−1^). *D*_Li_^+^ can be estimated by the slope of *I*_p_‐*v* ^0.5^ owing to the constants of *n, A*, and *C*_Li_^+^.

### Activation Energy (E_a_) Evaluation

The activation energy was calculated by the Arrhenius equation based on the temperature‐dependent EIS data.

(2) $$\frac{1}{R_{ct}}=Ae^{\left(\frac{-E_{a}}{RT}\right)}$$

where *R*_ct_ represents the interfacial resistance, *A* is the frequency factor, *R* denotes the gas constant (8.314 J mol^−1^ K^−1^), and *T* signifies the absolute temperature within the tests. *E*_a_ was obtained by calculating the slope of ln(1/*R*_ct_)‐1/*T*.

### DFT Calculations

All of the calculations were carried out with spin‐polarized density functional theory (DFT) method, as implemented with the Vienna ab initio simulation package. The electrons‐ion interactions are described by the Projector‐augmented wave potential. The exchange‐correlation functional is represented by the Perdew–Burke–Ernzerhof (PBE) functional within the generalized gradient approximation (GGA). The cutoff energy was set to 492 eV, and Monkhorst−Pack k‐meshes of 2 × 2 × 1 were set for the calculations. The Brillouin zone integrations were sampled by a Gamma point in k‐space based on Monkhorst–Pack scheme in the calculations. Vacuum layers of at least 10 Å were needed for non‐periodic directions. The force acting on every atom converged to 0.02 eV/Å, and the energy convergence criterion was less than 10^−5^ eV. The polysulfide species, the SPAN molecules, and the adsorption system were respectively simulated in a cubic box with side lengths of 25 Å. The DFT‐D3 semi‐empirical correction was described via Grimme's scheme method. Climbing‐image nudged elastic band (CI‐NEB) method was used to find the minimum energy path and energy barrier of Li_2_S dissociation on In_5_‐SPAN and In_0_‐SPAN.

The adsorption energies (*E*_ads_) of adsorbate on the substrate are defined as:

(3) $$E_{\mathrm{ads}}=E_{\mathrm{sur}+\mathrm{mol}}-E_{\mathrm{sur}}-E_{\mathrm{mol}}$$

where *E*_sur  +  mol_ represents the total energy of adsorption system. *E*_sur_ is the total energy of the catalyst substrate, *E*_mol_ represents the total energy of adsorbate, respectively. According to the formula, the negative values of *E*_ads_ suggest spontaneous and exothermal, while the positive values represent the opposite.

## Conflict of Interest

The authors declare no conflict of interest.

## Author Contributions

C.H. and Y.G. contributed equally to this work. C. H. designed and performed the experiment, analyzed the data, and drafted the original manuscript under the supervision of Z.Z. Y.G. performed the XAFS experiments and analyzed the resulting data, and co‐wrote the manuscript. Q.Z. and M.X. conducted electrochemical measurements and processed the electrochemical data. K.Y. and W.Z. assisted with data interpretation and contributed to the critical discussion of the redox mechanism. Y.G., S.R.P.S., K.Y., and Z.Z. conceived and supervised the project and provided critical revisions. All authors reviewed and approved the final version.

## Supporting information



Supporting Information

## Data Availability

The data that support the findings of this study are available from the corresponding author upon reasonable request.
