# Supplementary material for: Reconfigurable In–S Coordination in SPAN Cathodes: Unlocking High Sulfur Utilization and Fast Kinetics for Practical Li‒S Batteries
Source: Adv Sci (Weinh). 2025 Jul 30;12(40):e07385. doi: 10.1002/advs.202507385 (PMC12561284; doi:10.1002/advs.202507385)
Supplement: Supplementary file 1 — Supporting Information [file ADVS-12-e07385-s001.docx]

Supporting information

**Reconfigurable In‒S Coordination in SPAN Cathodes: Unlocking High Sulfur Utilization and Fast Kinetics for Practical Li‒S Batteries**

Cheng Huang^⸶^, Yi Gong^⸶^, Qi Zhu, Miaoran Xu, Kai Yang^*^, José V. Anguita, Wei Zhang, S. Ravi P. Silva^*^, Yanfeng Gao^*^ and Zongtao Zhang^*^


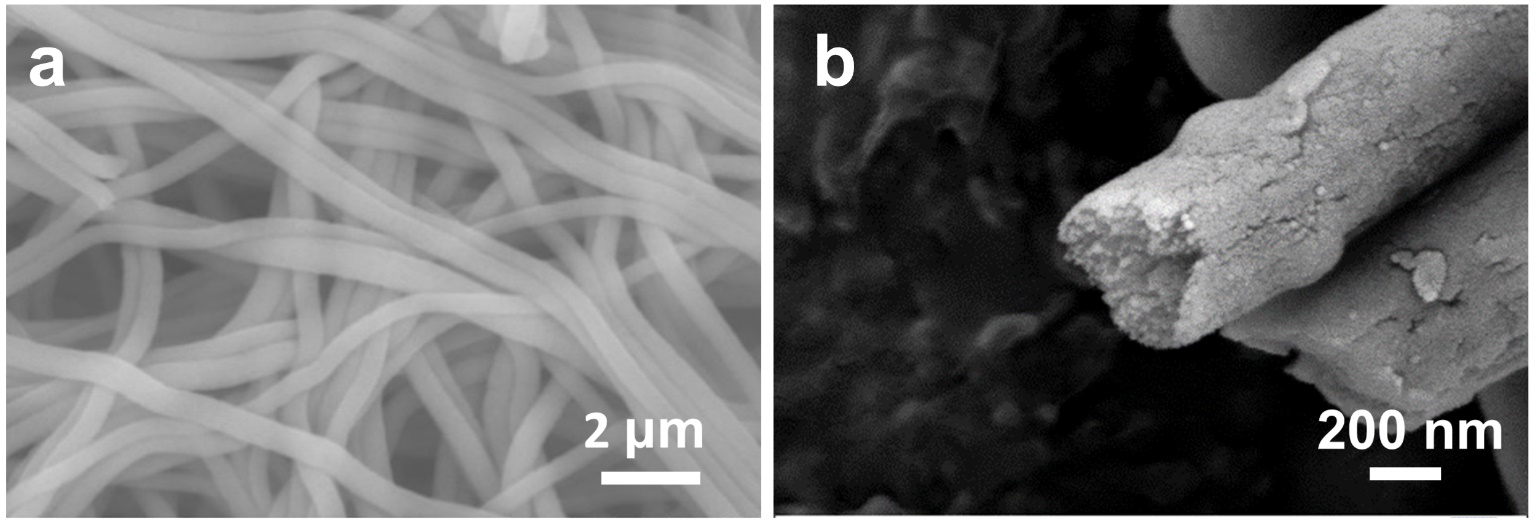


Figure S1. SEM images of In_5_-PAN fibers.


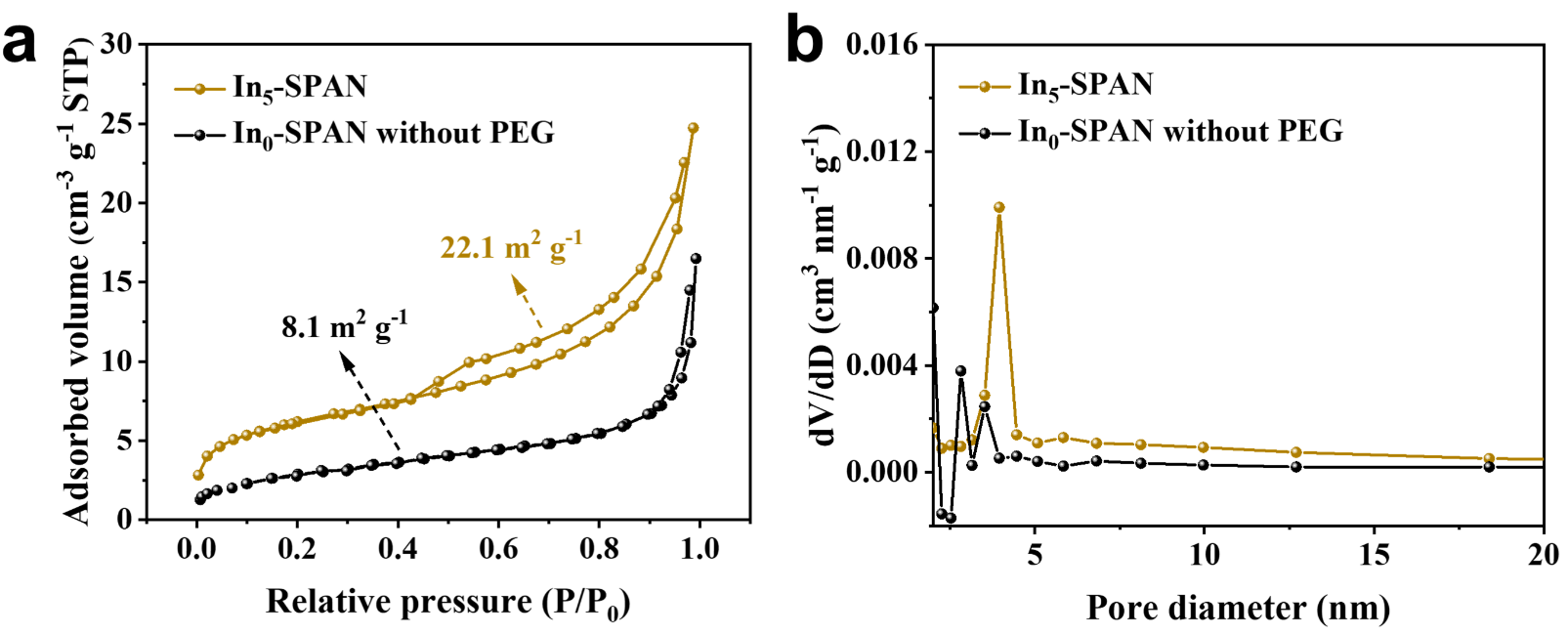


Figure S2. N_2_ sorption isotherms a) and corresponding pore diameter distributions b) of In_5_-SPAN fibers and In_0_-SPAN fibers without PEG.


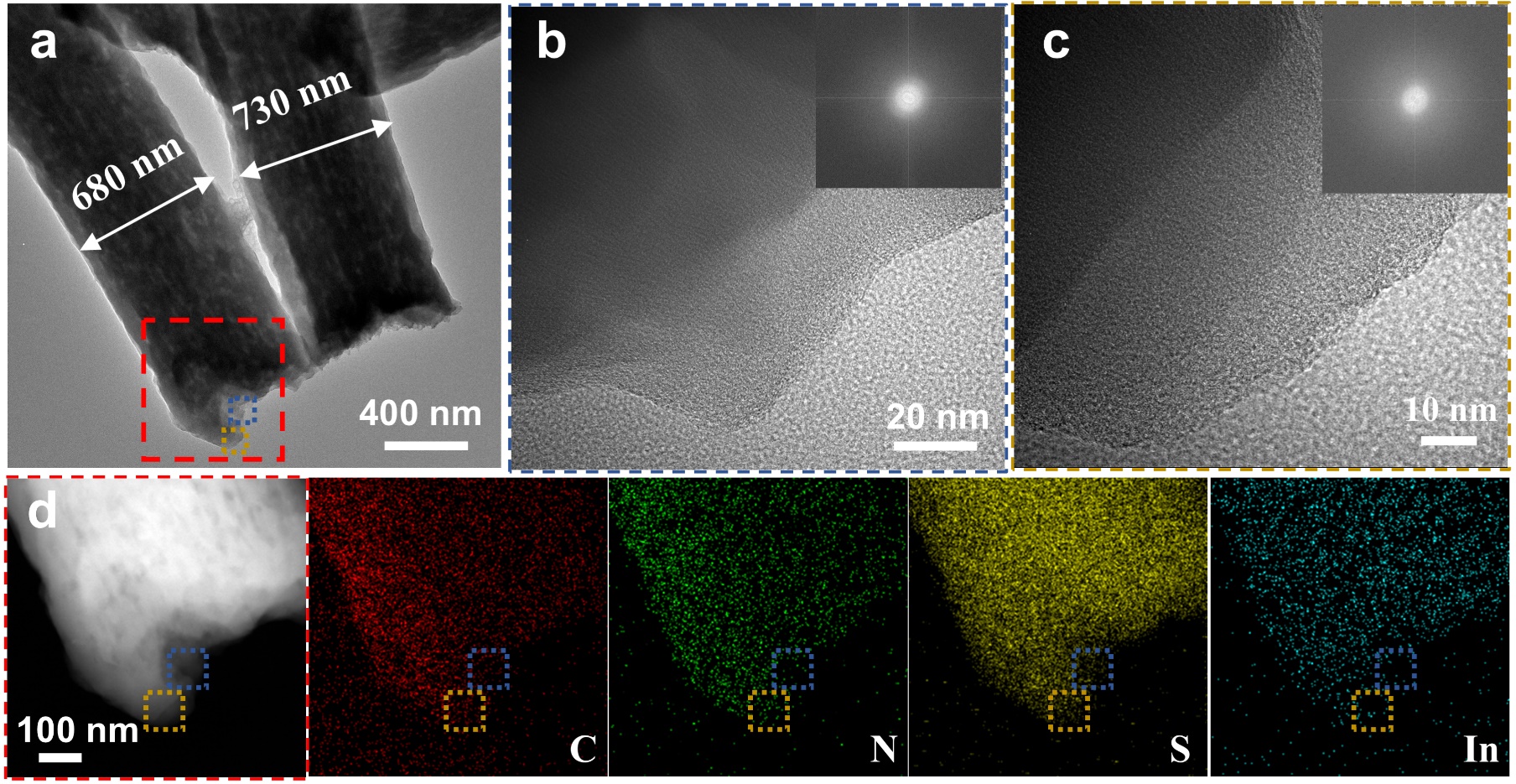


Figure S3. a) High-resolution TEM image of In_5_-SPAN fibers, b) and c) amplified images of fibers within the blue and brown rectangles, respectively. d) dark-field TEM image, and corresponding elemental mapping of In_5_-SPAN.


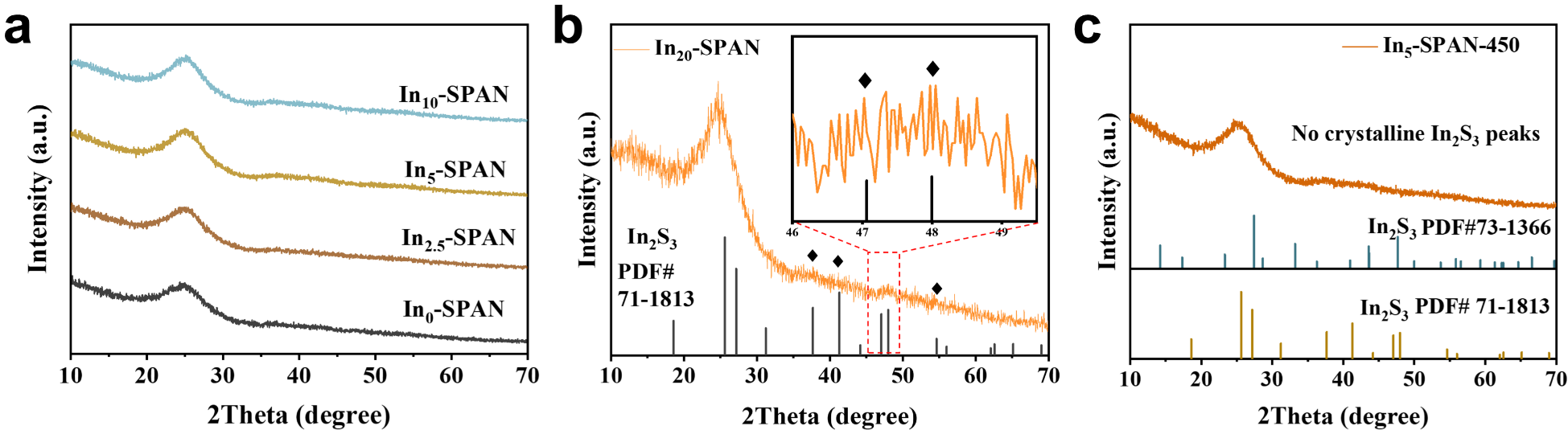


Figure S4. XRD patterns of a) In_x_-SPAN, b) In_20_-SPAN (Inset is the amplified image of the red rectangle) and c) In_5_-SPAN 450 ℃.

Figure S5. XRD pattern of the In_2_S_3_ composite. The In_2_S_3_ composite is synthesized using indium acetate as the precursor under the same preparation conditions with the In_5_-SPAN fibers.


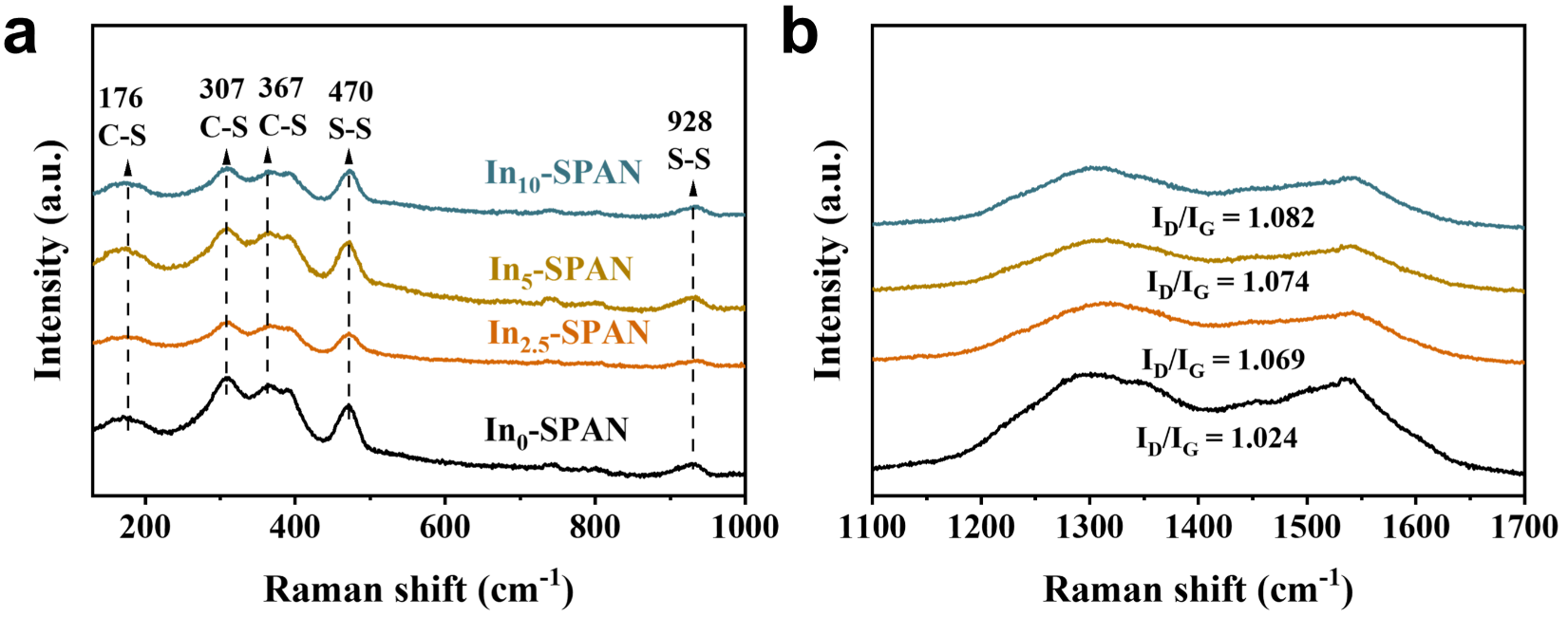


Figure S6. Raman spectra of In_x_-SPAN.

Figure S7. XPS full spectrum of In_5_-SPAN.


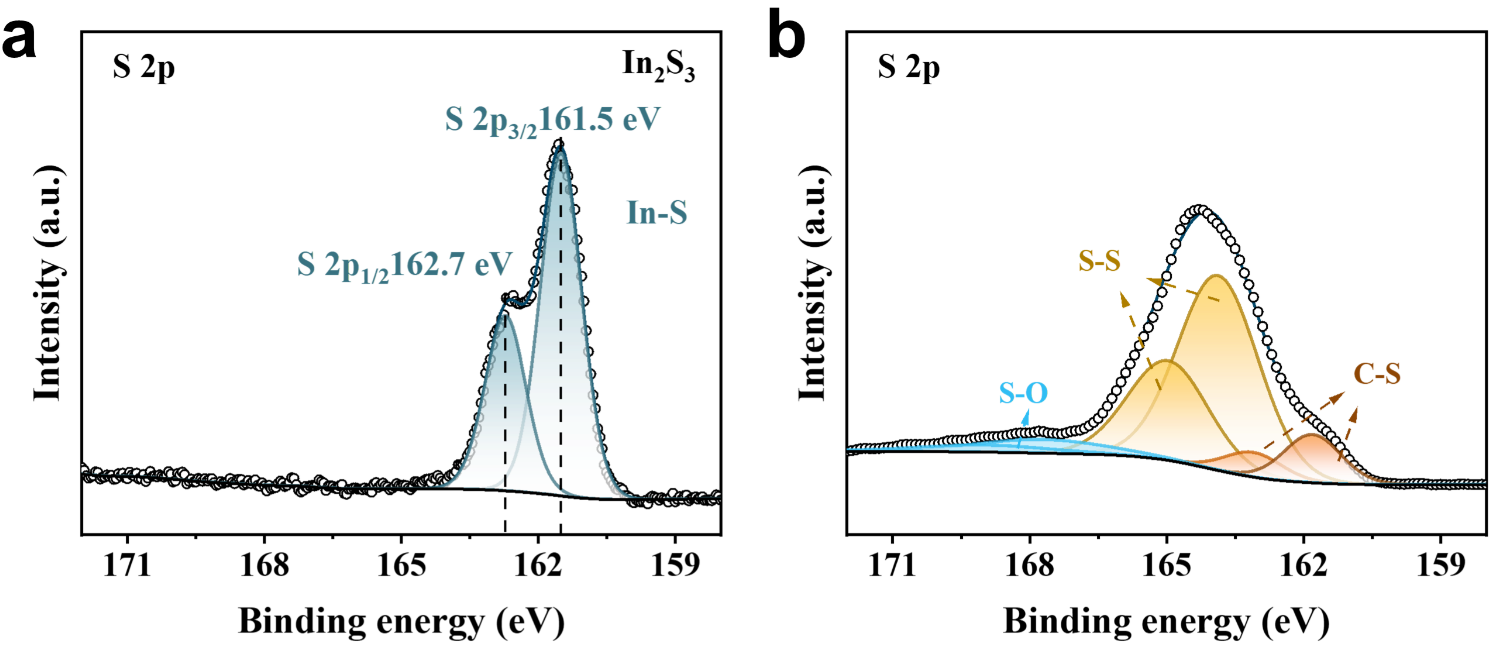


Figure S8. S 2p XPS spectrum of a) In_2_S_3_ and b) In_0_-SPAN.

Figure S9. WT-EXAFS contour plot of In_2_O_3_.


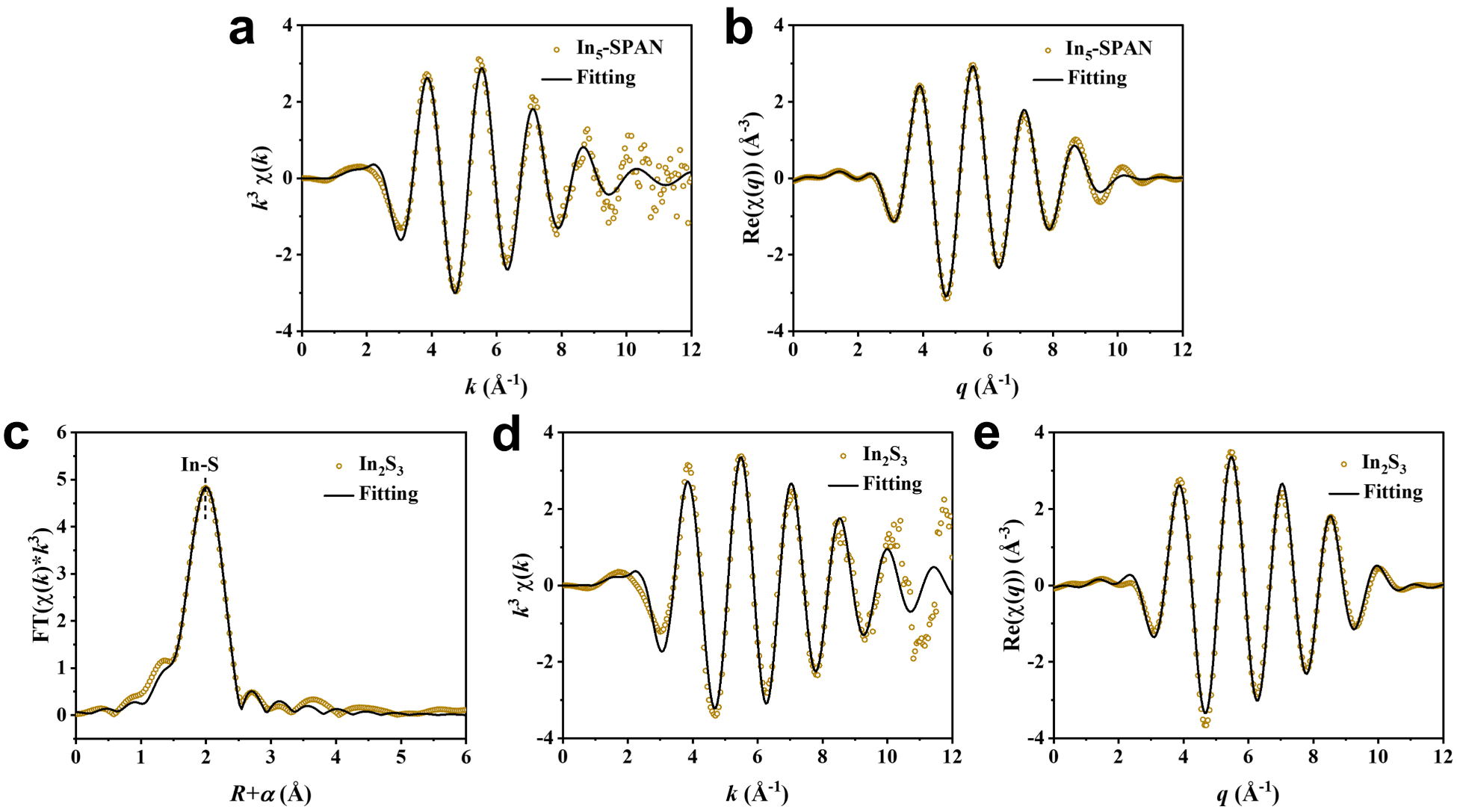


Figure S10. The fitting curves of In_5_-SPAN: a) EXAFS; b) Inverse FT-EXAFS; The fitting curves of In_2_S_3_: c) FT-EXAFS; d) EXAFS; e) Inverse FT-EXAFS.


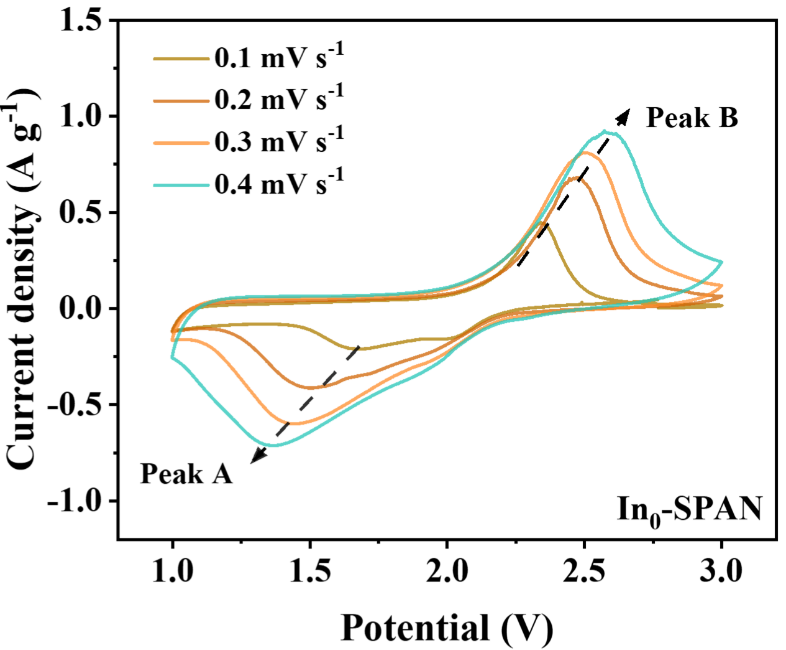


Figure S11. CV profiles of In_0_-SPAN cathode at different scan rates.


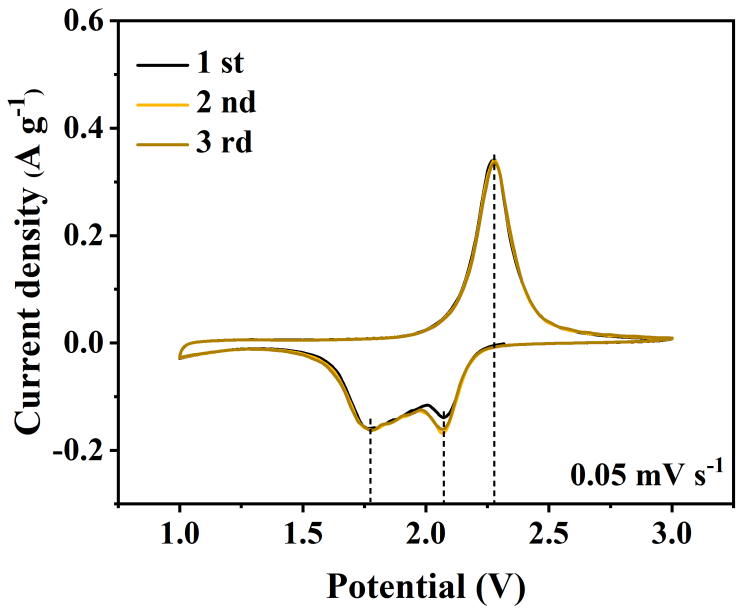


Figure S12. CV profiles of In_5_-SPAN cathode at 0.05 mV s^-1^.


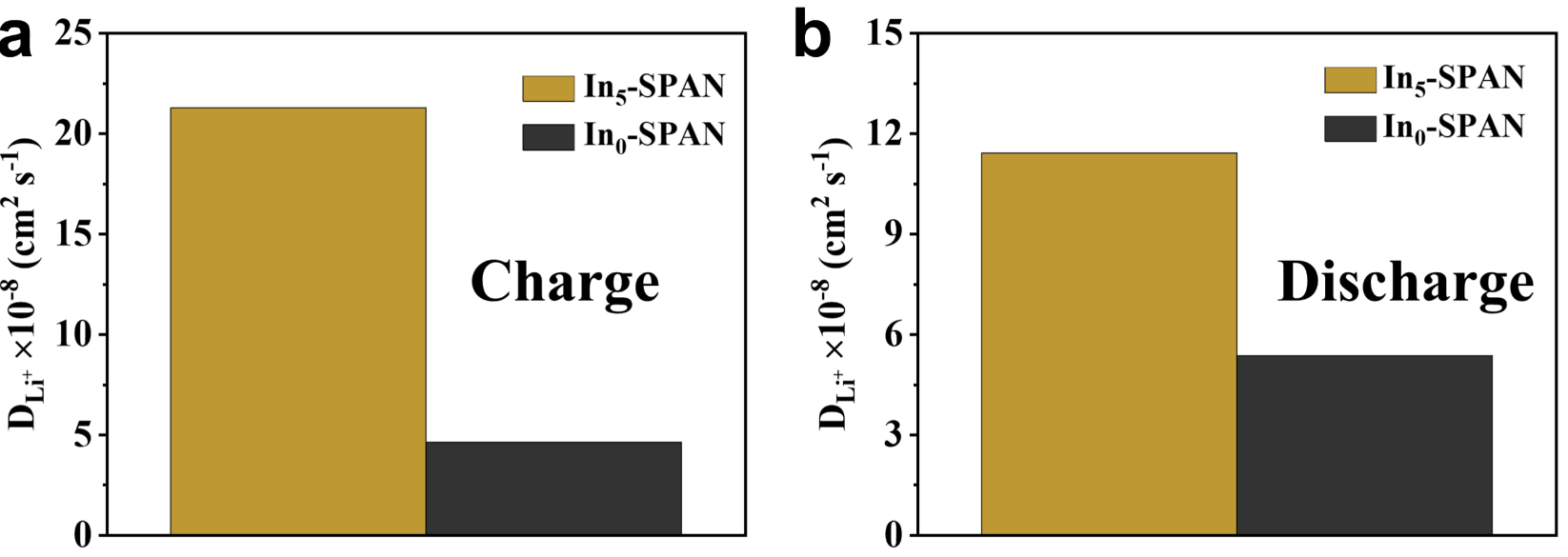


Figure S13. Calculated Li^+^ diffusion coefficient of oxidation peak a) and reduction peak b) of In_5_-SPAN and In_0_-SPAN cathodes.

Figure S14. The discharge curve of the In_5_-SPAN electrodes at the second cycle, the cell was discharged to the designated voltage marked with black dots, and then subjected to EIS tests at different temperatures immediately.


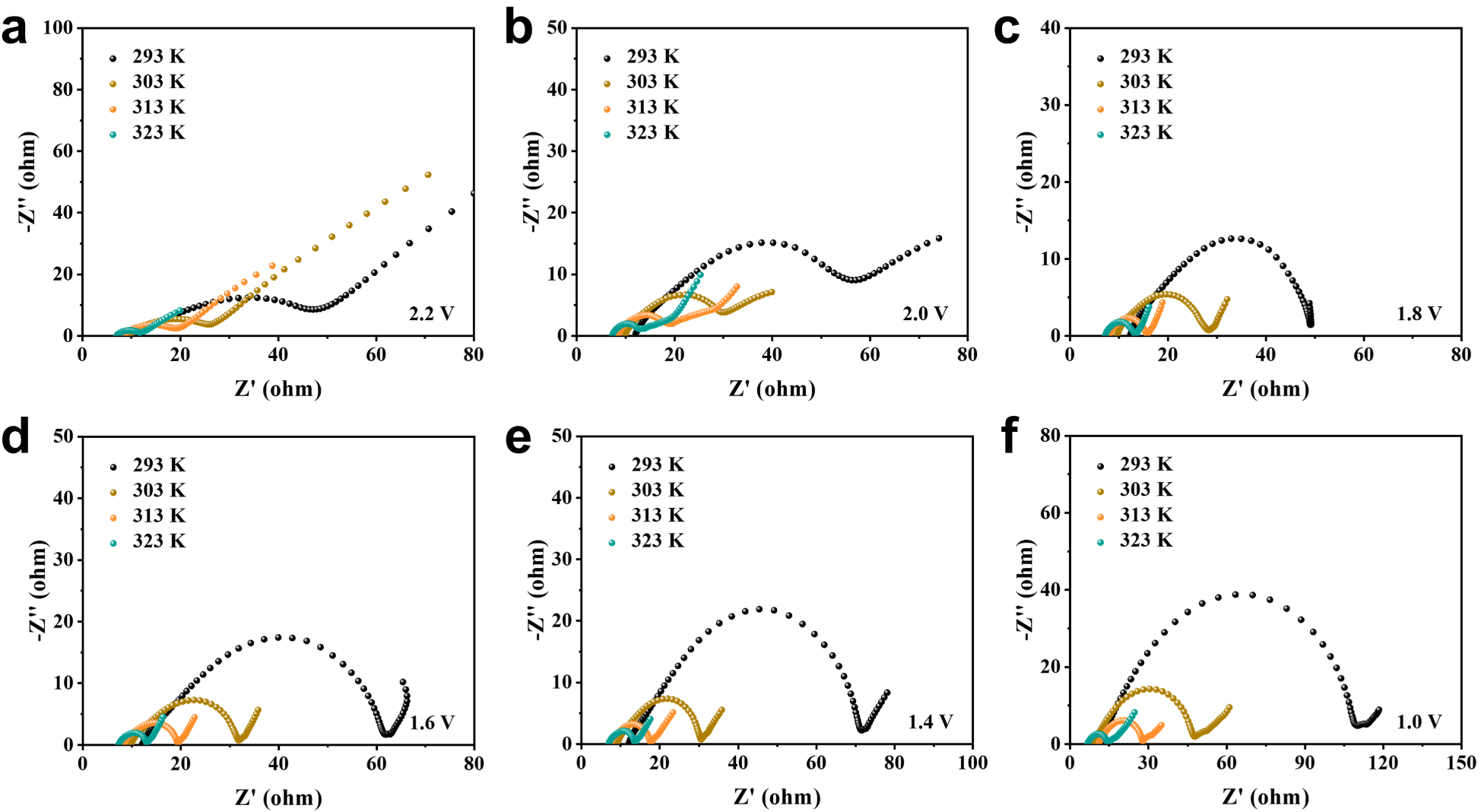


Figure S15. a-f) EIS curves of the In_0_-SPAN electrodes at various voltages and temperatures.


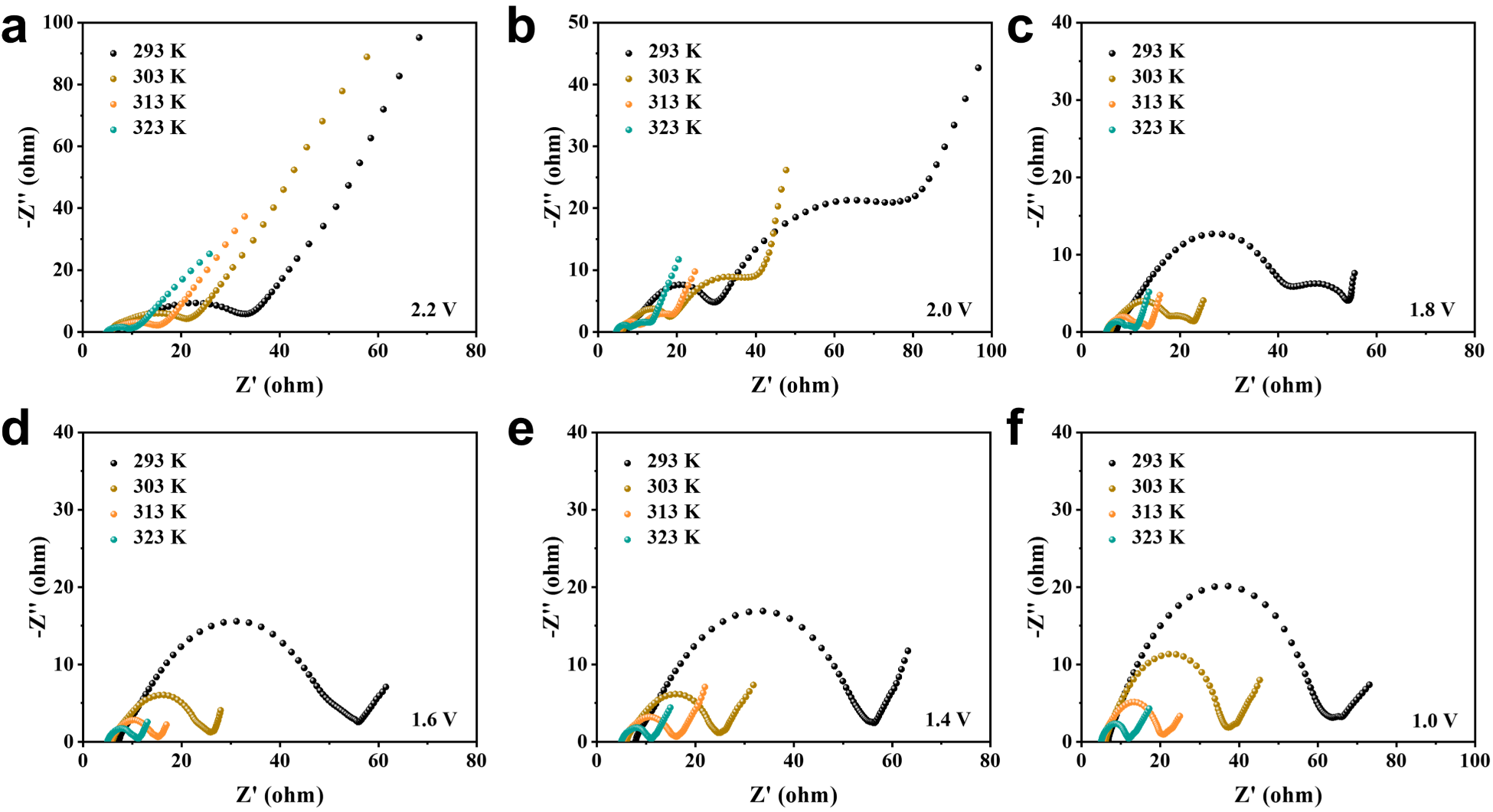


Figure S16. a-f) EIS curves of the In_5_-SPAN electrodes at various voltages and temperatures.


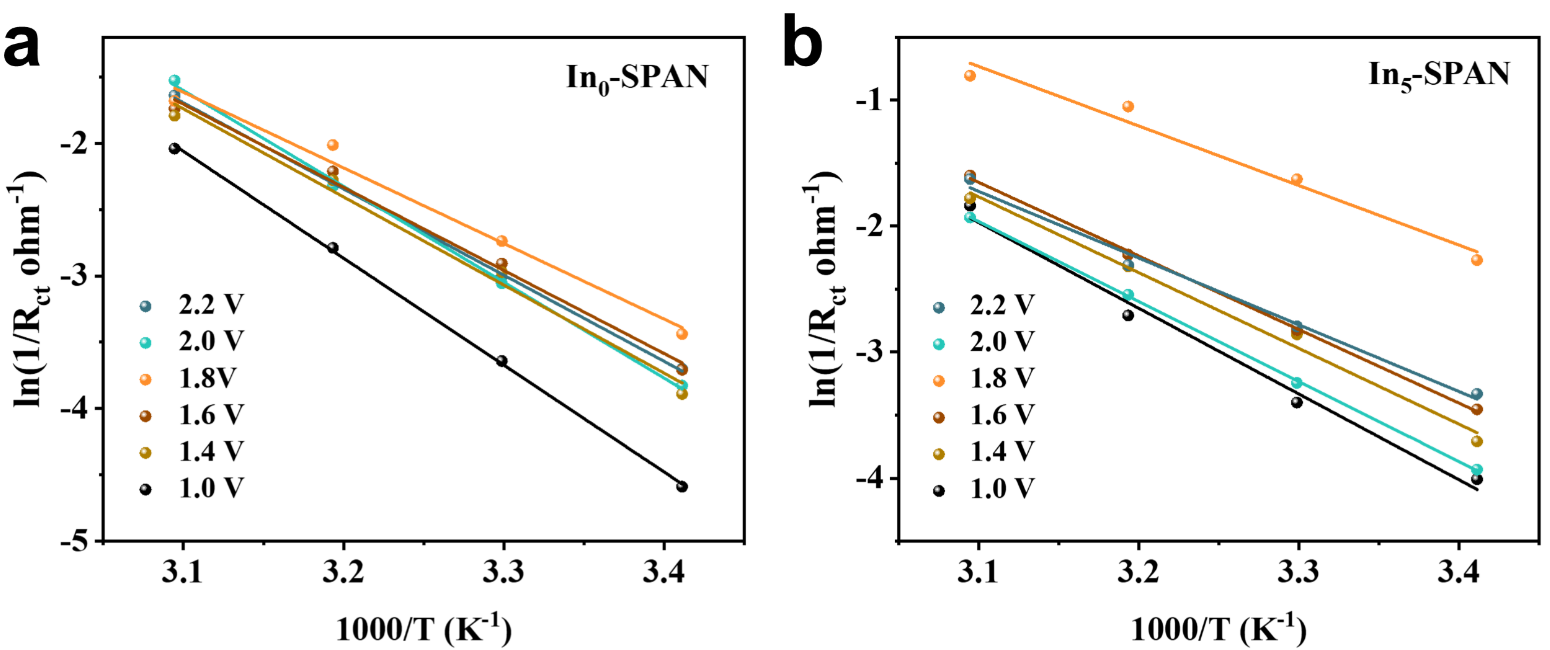


Figure S17. Arrhenius plots for the charge transfer resistance (*R*_ct_) of a) In_0_-SPAN and b) In_5_-SPAN electrodes.


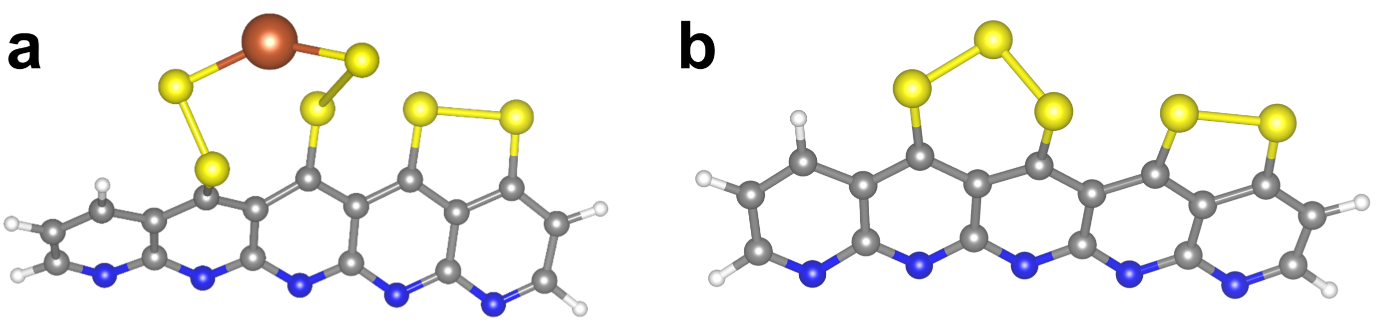


Figure S18. Optimized structure of a) In_5_-SPAN and b) In_0_-SPAN, respectively.


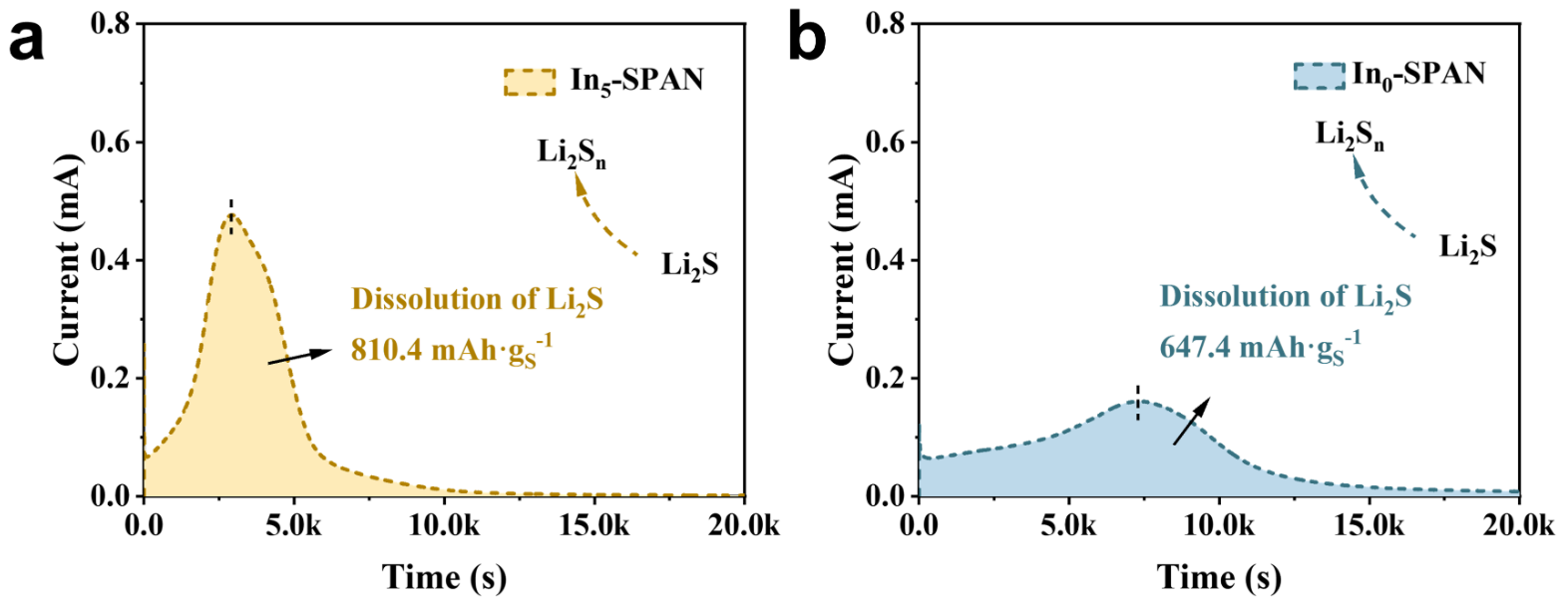


Figure S19. Potentiostatic charge profiles of Li_2_S for a) In_5_-SPAN and b) In_0_-SPAN.


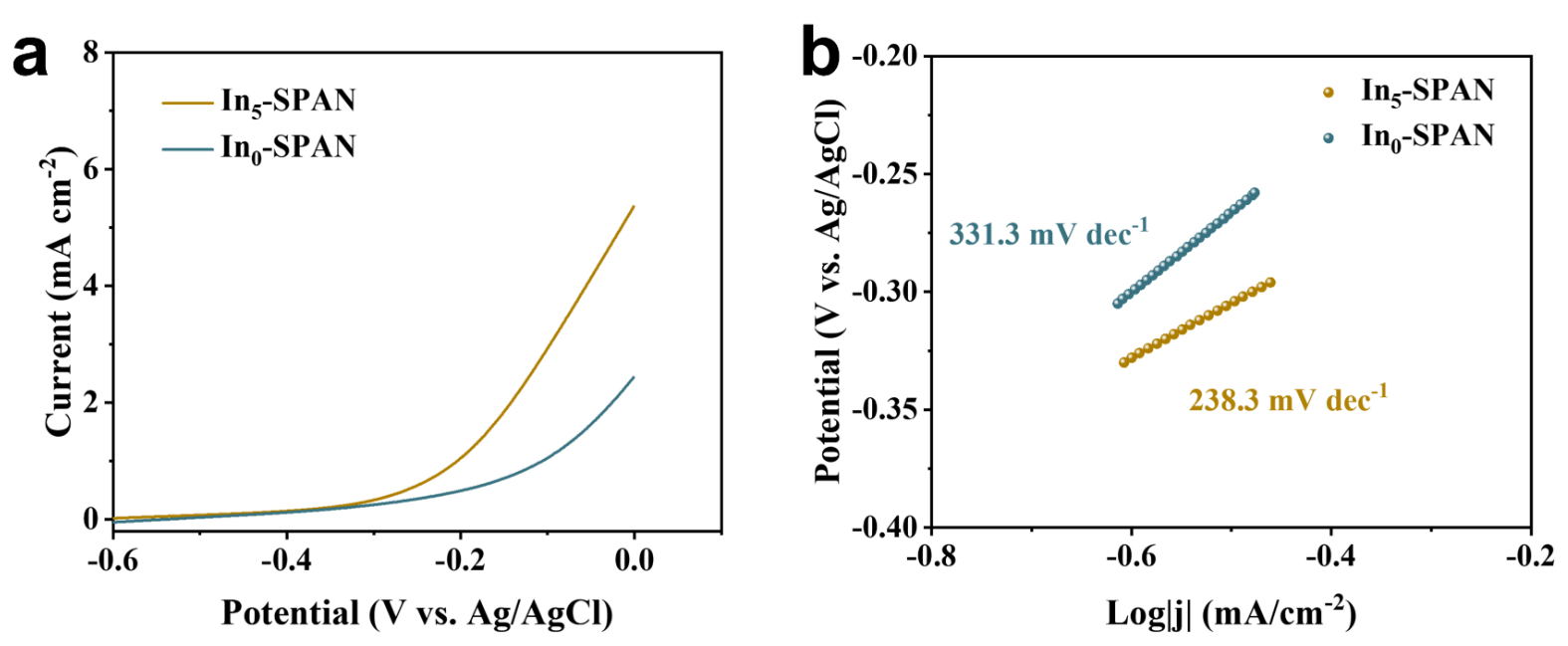


Figure S20. a) Linear sweep voltammetry curves of Li_2_S oxidation and b) related Tafel plots on In_5_-SPAN and In_0_-SPAN surfaces.


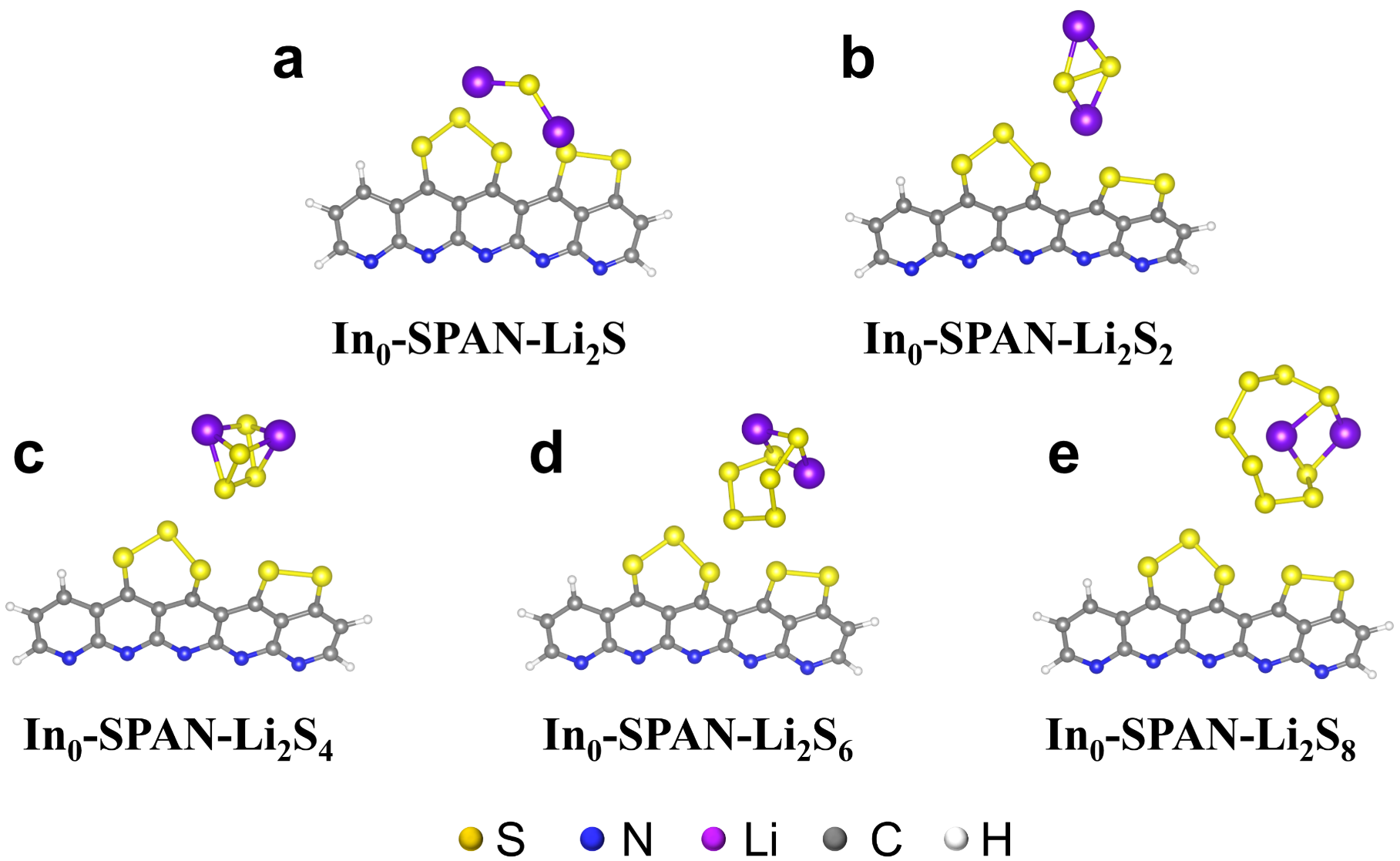


Figure S21. Optimized geometries of different lithium polysulfides adsorbed on In_0_-SPAN, obtained from DFT calculations.


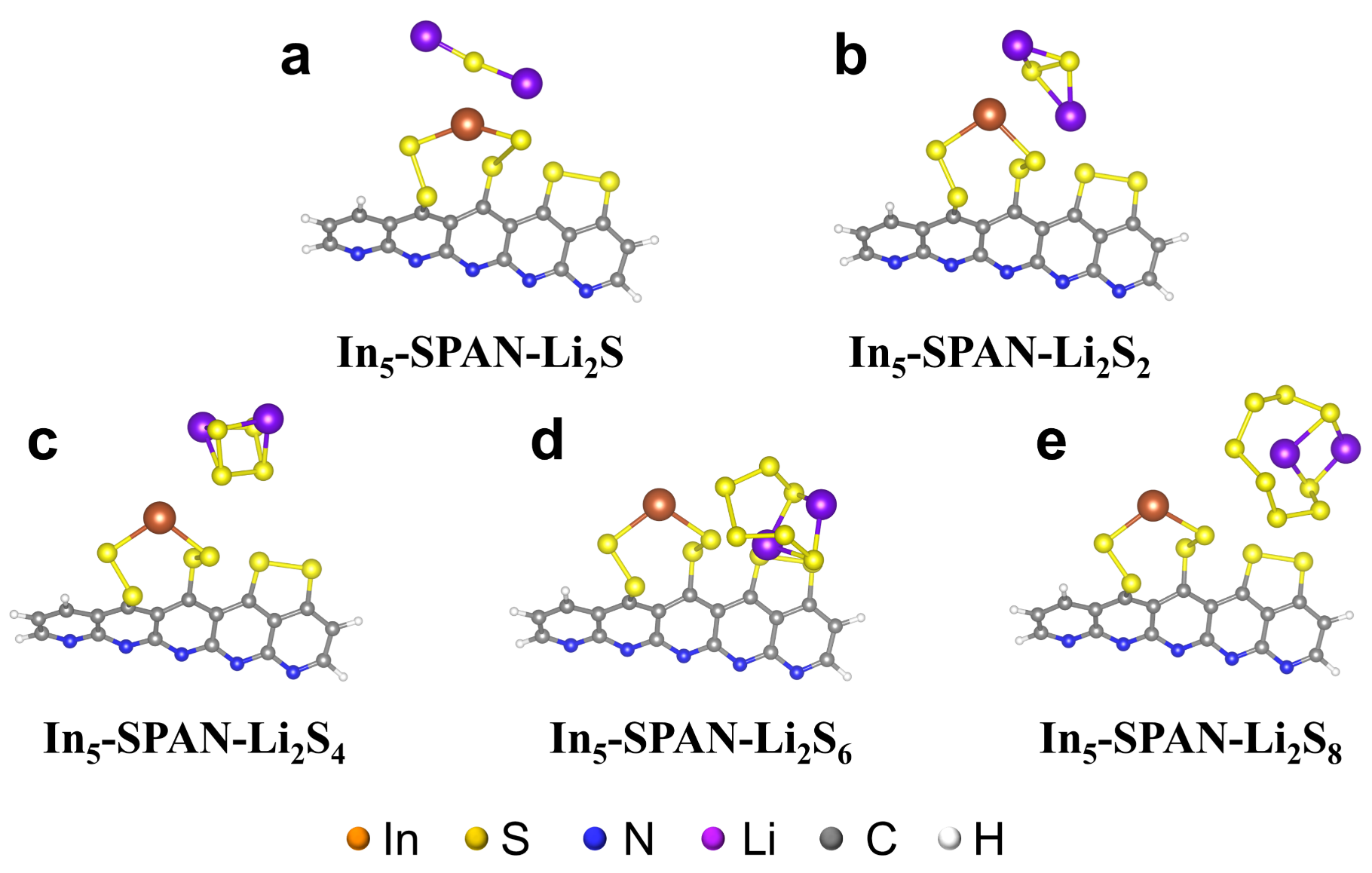


Figure S22. Optimized geometries of different lithium polysulfides adsorbed on In_5_-SPAN, obtained from DFT calculations


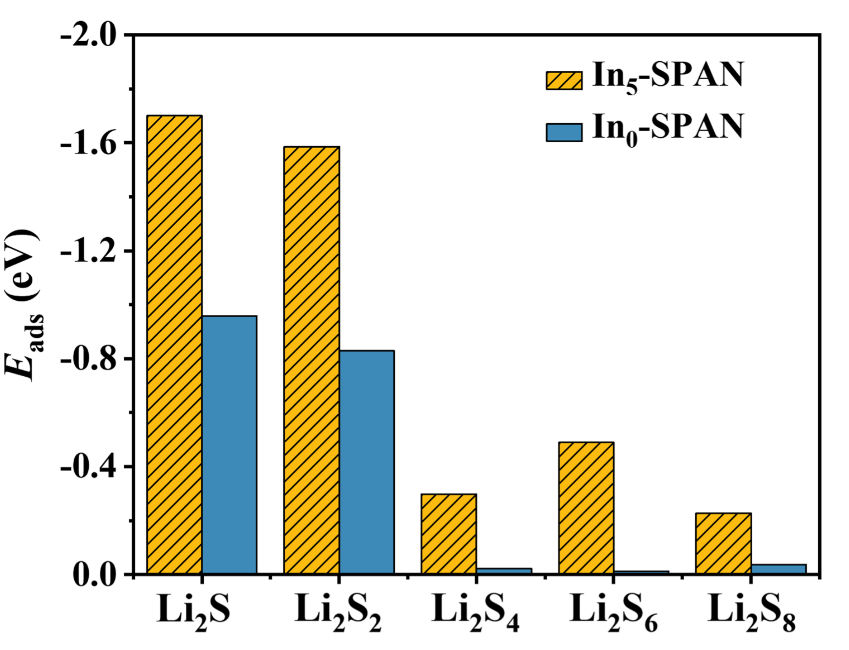


Figure S23. Calculated adsorption energies between different lithium polysulfides and In_5_/In_0_-SPAN cathodes.


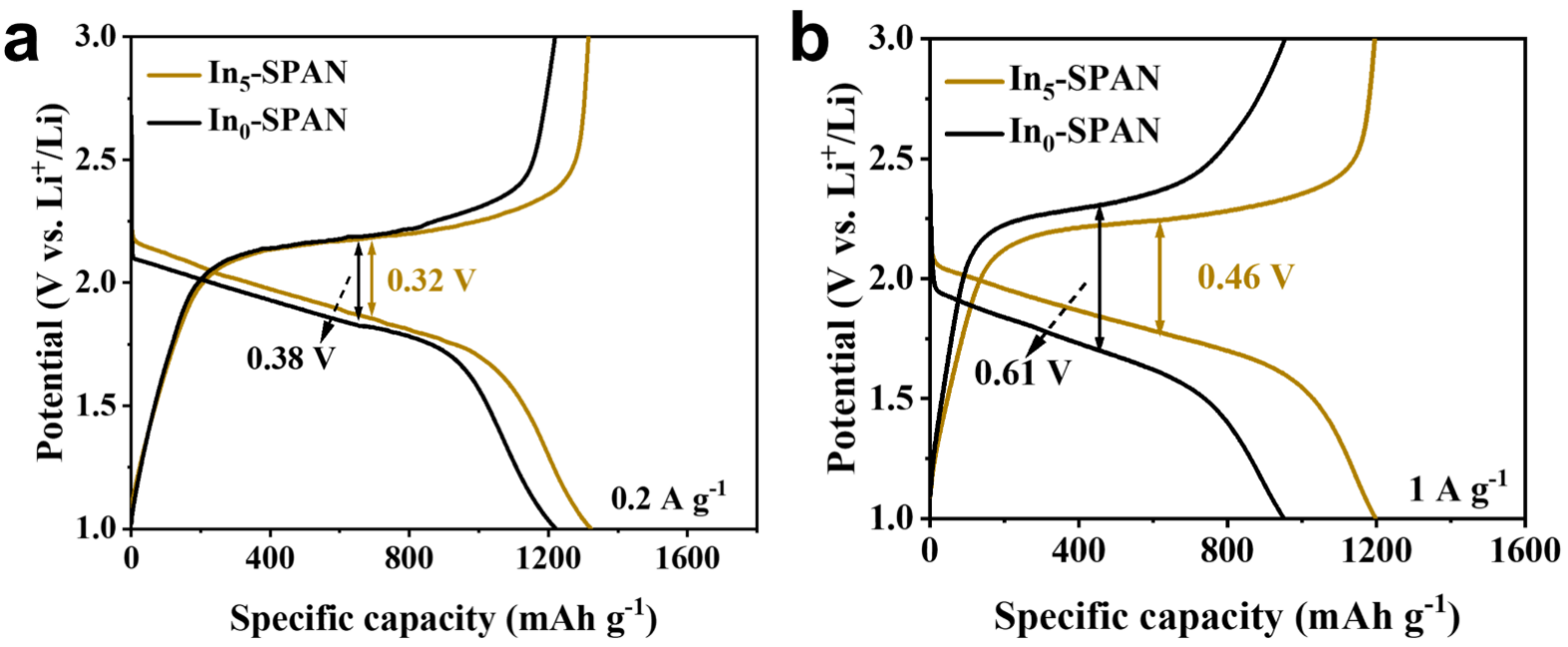


Figure S24. Electrochemical charge and discharge profiles of In_5_-SPAN and In_0_-SPAN cathode at a) 0.2 A g^-1^ and b)1 A g^-1^.

Figure S25. Cycle performance comparison of In_x_-SPAN cathode at 0.5 A g^-1^.


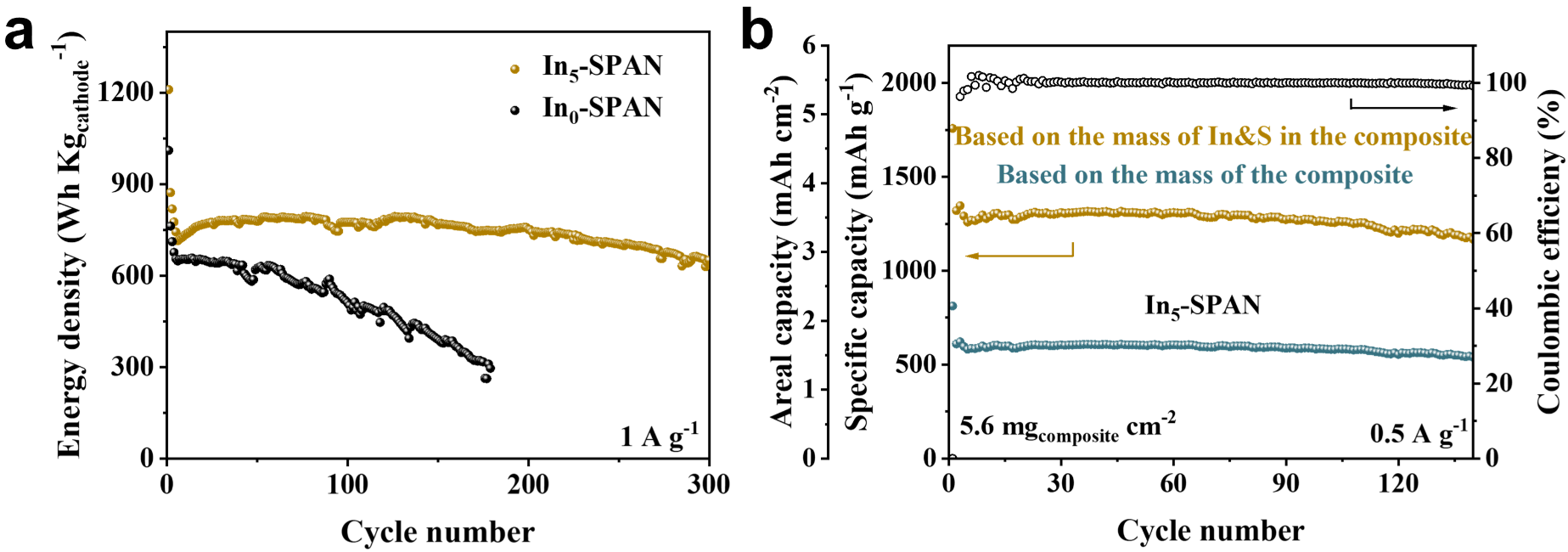


Figure S26. Cycle performance of In_5_-SPAN and In_0_-SPAN cathodes: a) Gravimetric energy density comparisons at 1 A g^-1^. b) Cycle performance of In_5_-SPAN cathode with the mass loading of 5.6 mg_span_ cm^-2^ at 0.5 A g^-1^.

The gravimetric discharge energy density of the In_5_-SPAN and In_0_-SPAN cathodes was determined according to the total mass of the cathode materials. Given that the coordination number of In atoms in the In_5_-SPAN composite is marginally higher than that in crystalline In_2_S_3_, the reversible capacity contributions from In-S bonds within In_5_-SPAN were quantitatively evaluated by standardizing against an equivalent molar quantity of crystalline In_2_S_3_. The theoretical capacity of crystalline In_2_S_3_ was calculated to be 493.6 mAh g^-1^.

$$Gravimetric Energy Density (Wh {kg}^{-1})= \frac{Operating {Voltage}^{*} \left( V \right)\times Capacity (Ah)}{Mass of the cathode (kg)}$$

^*^Average operating voltage was taken as 1.8 V for SPAN based on the reports^1, 2^.


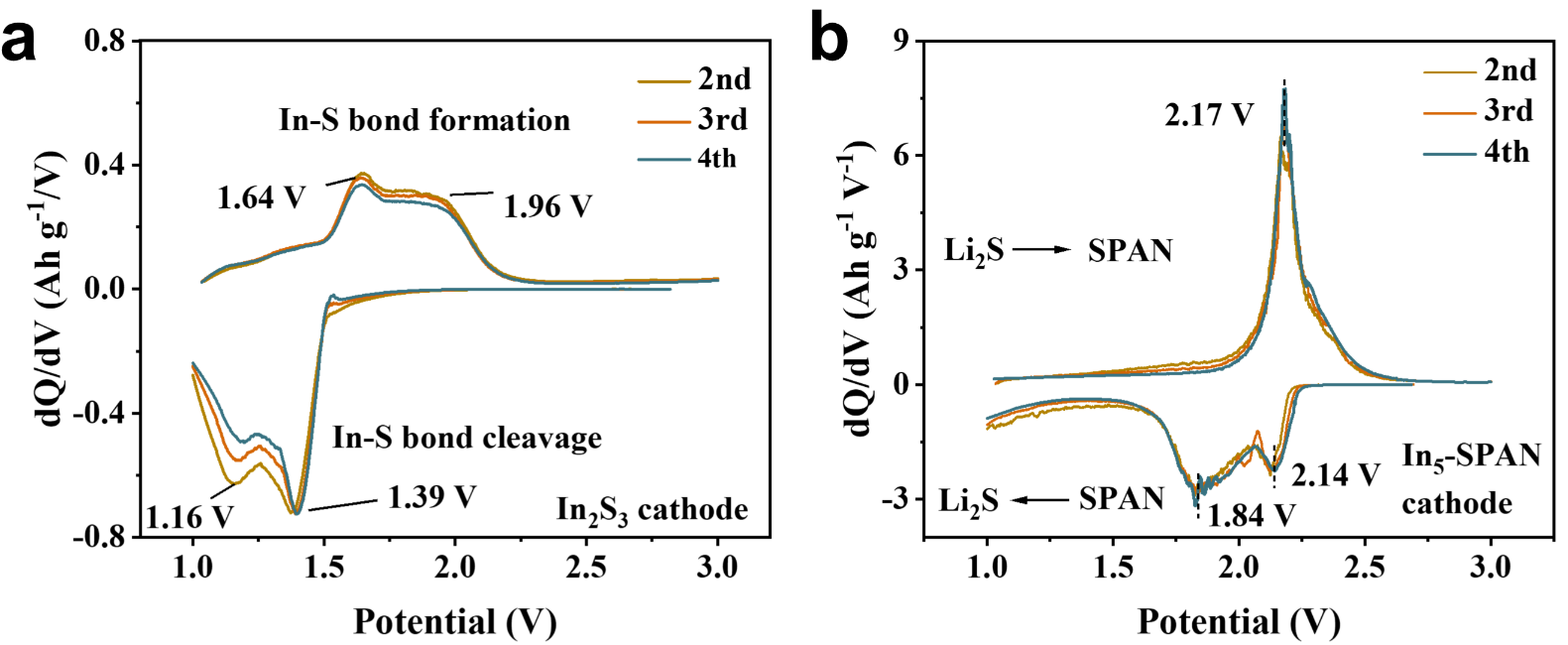


Figure S27. dQ/dV curves of a) In_2_S_3_ cathode, b) In_5_-SPAN cathode and c) In_0_-SPAN cathode.


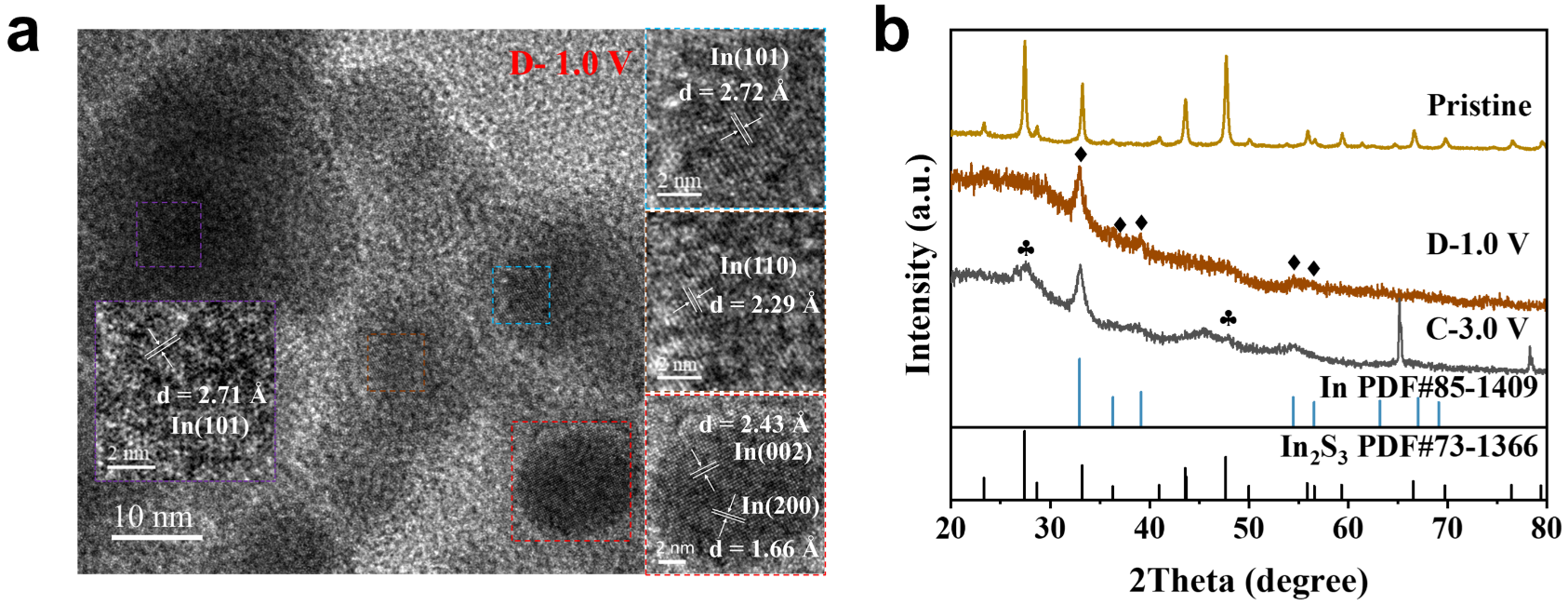


Figure S28. a) TEM image and corresponding amplified images at fully discharged state. b) XRD patterns of In_2_S_3_ cathode at pristine, fully discharged and fully charged state.


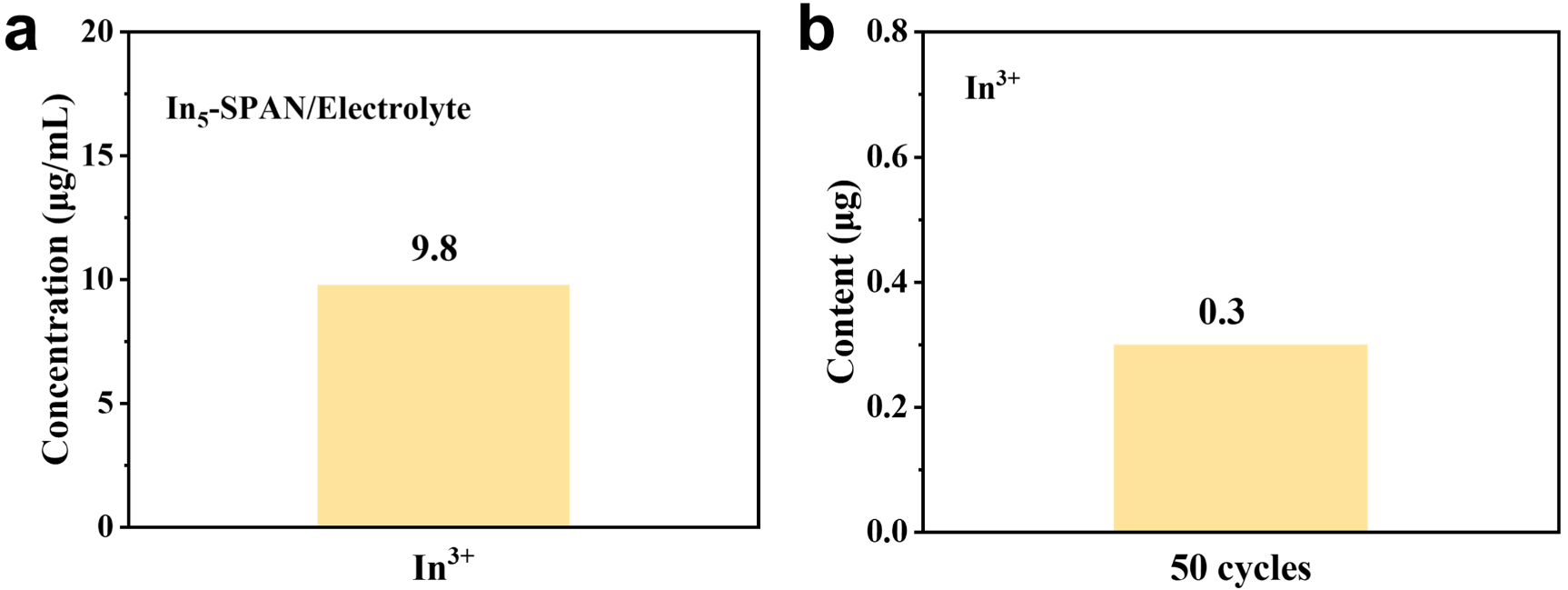


Figure S29. a) The dissolved In^3+^ concentration of In_5_-SPAN. The In_5_-SPAN composite is mixed with ether electrolyte for one day and then performed with ICP-OES tests. b) The dissolved In^3+^ content of the coin cells after 50 cycles.


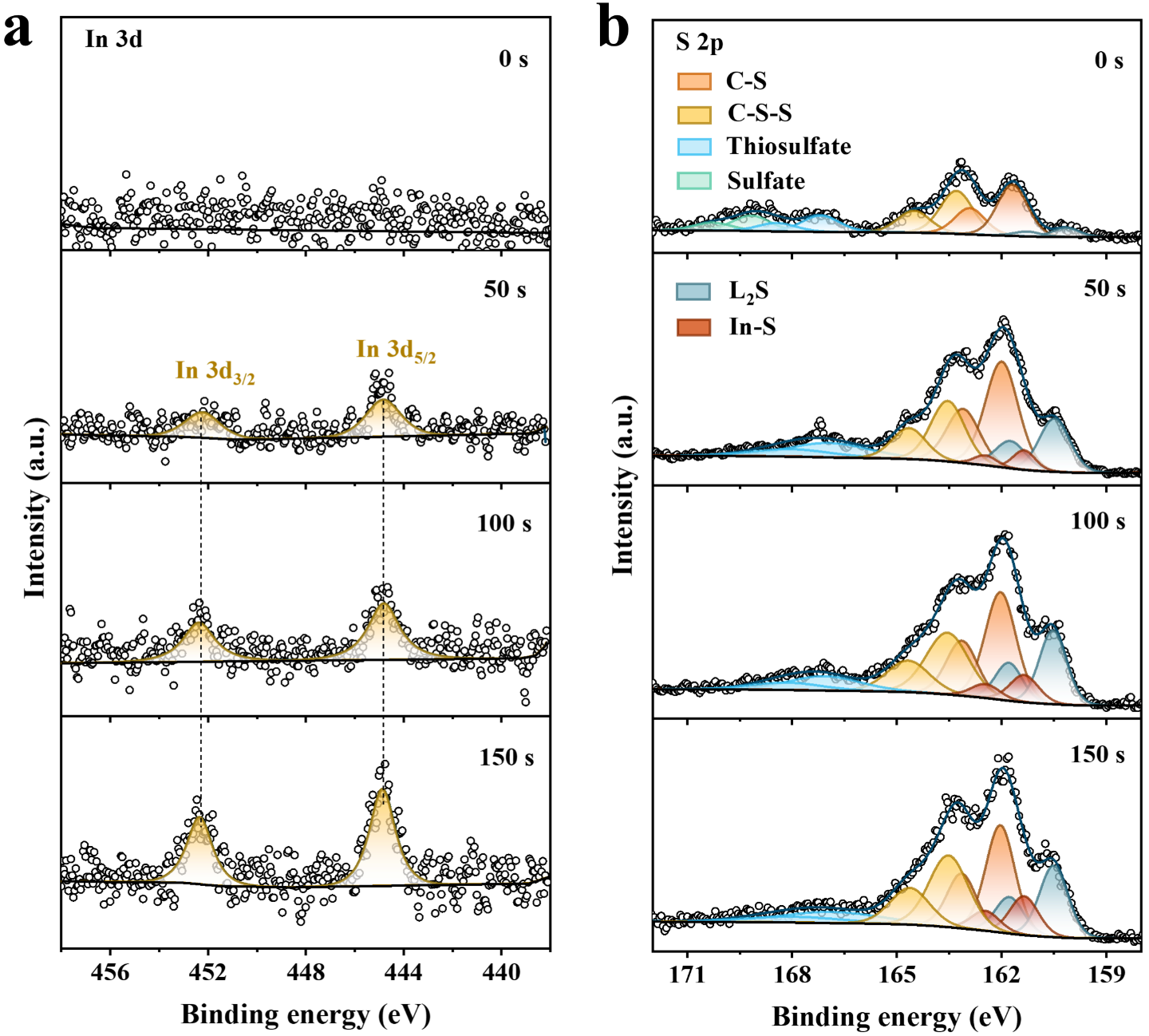


Figure S30. a) In 3d and b) S 2p XPS spectrum of the In_5_-SPAN cathodes after 50 cycles under the linearly increasing etch time, each 50 s increase in etch time corresponds to a 10 nm deeper etch.


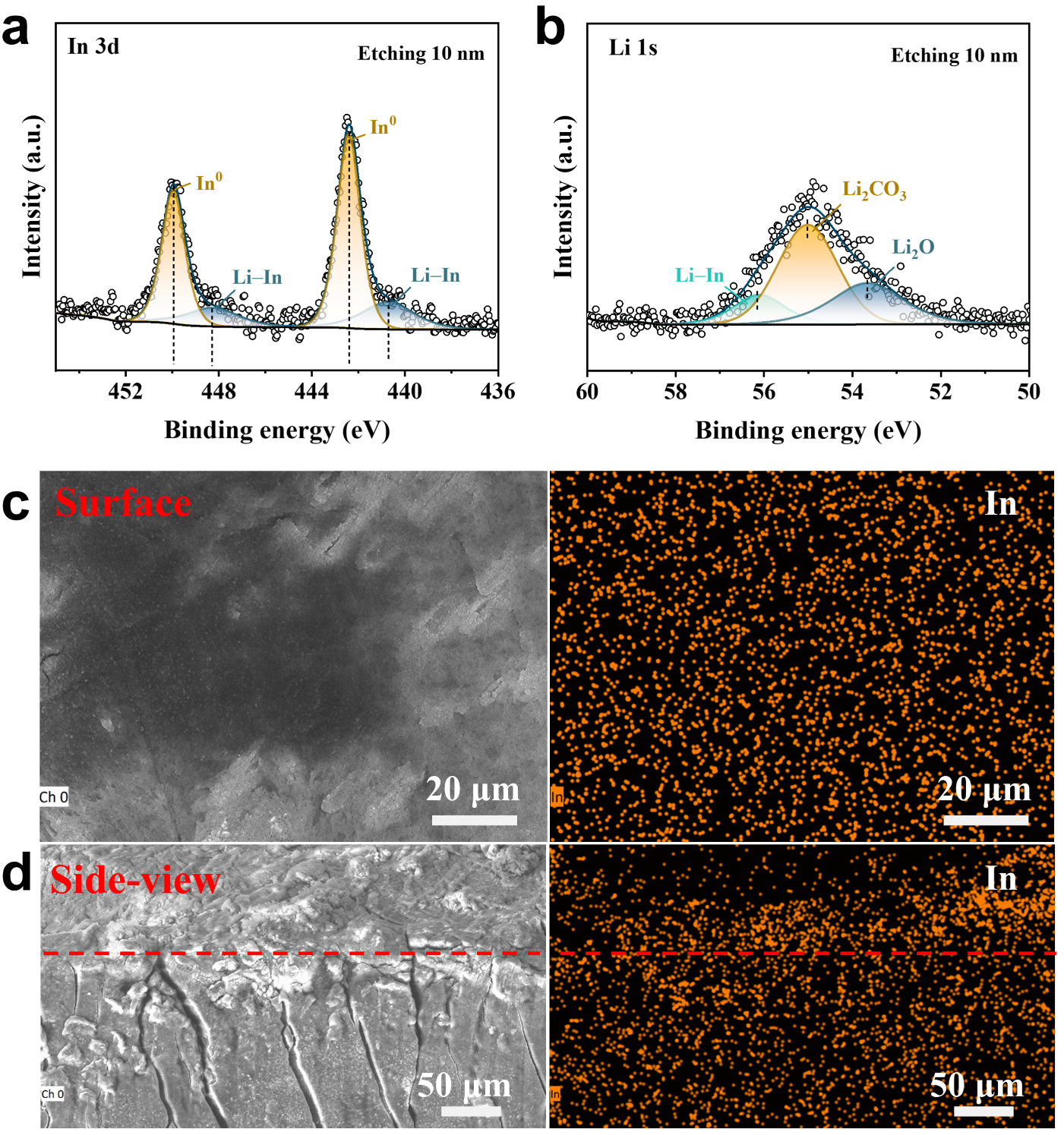


Figure S31. a) In 3d and b) Li 1s XPS spectra of the lithium anode. c) Surface and d) cross-section SEM images of the lithium anode and corresponding EDS mapping of In (based on In_5_-SPAN cathode, SPAN loading: 8.7 mg_span_ cm^-2^).


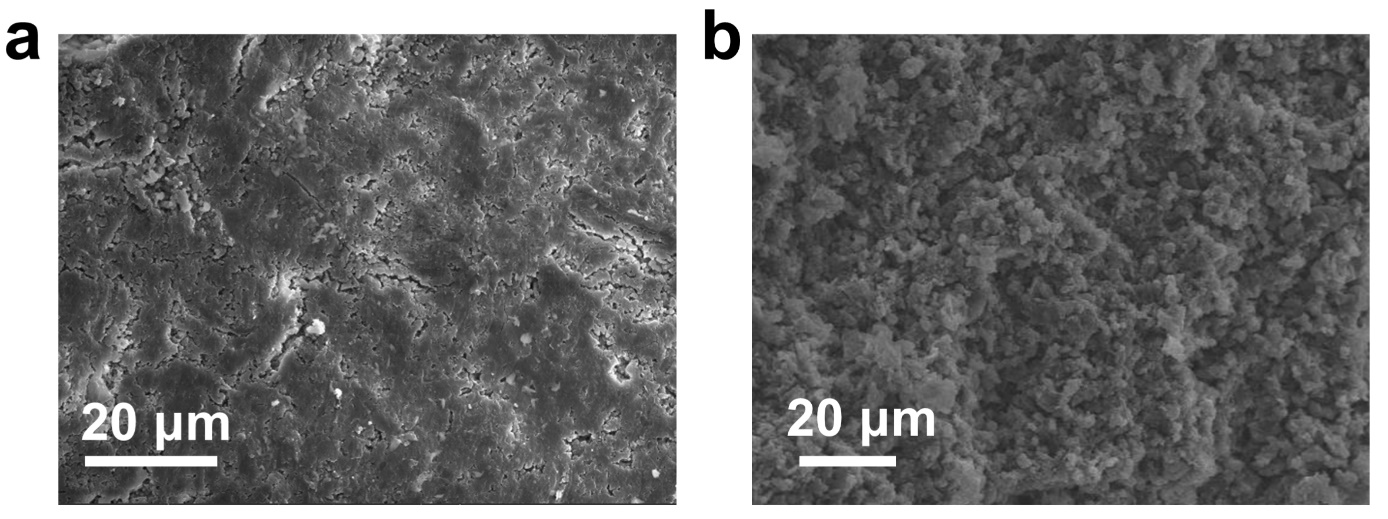


Figure S32. SEM images of anode based on a) In_5_-SPAN cathode and b) In_0_-SPAN cathode after 30 cycles. (SPAN loading: 8.7 mg_span_ cm^-2^, E/SPAN = 4.1).

**Table S1. The C, N, S and H content in the composite based on the elemental analysis and the In content in the composite based on the ICP-OES.**

| **Materials** | **C (wt%)** | **N (wt%)** | **S (wt%)** | **H (wt%)** | **In (wt%)** |
| --- | --- | --- | --- | --- | --- |
| SPAN powder | 40.91 | 14.45 | 38.52 | 0.936 | 0 |
| SPAN fiber | 38.51 | 13.95 | 40.87 | 0.843 | 0 |
| In_0_-SPAN | 38.15 | 13.39 | 44.17 | 0.592 | 0 |
| In_2.5_-SPAN | 38.26 | 12.48 | 45.04 | 0.566 | 0.6 |
| In_5_-SPAN | 37.44 | 12.86 | 46.20 | 0.485 | 1.18 |
| In_10_-SPAN | 36.67 | 12.81 | 45.24 | 0.465 | 2.36 |

**Table S2. The theoretical specific capacity calculation of In_x_-SPAN based on the active material and the composite.**

| **Materials** | **In&S (wt%) in the composite** | **Theoretical specific capacity (mAh g^-1^, based on In and S)** | **Theoretical specific capacity (mAh g^-1^, based on the composite)** |
| --- | --- | --- | --- |
| In_0_-SPAN | 44.17 | 1635.4 | 739.8 |
| In_2.5_-SPAN | 45.64 | 1653.0 | 754.4 |
| In_5_-SPAN | 47.38 | 1633.1 | 773.8 |
| In_10_-SPAN | 47.58 | 1591.4 | 757.5 |

**Table S3. The assignments of FTIR peaks of In_5_-SPAN.**

| **Wavenumber (cm^-1^)** | **assignments** |
| --- | --- |
| 1500 | C=C symmetric stretch |
| 1428 | C=N asymmetric stretch |
| 1360 | C=C deformation |
| 1245 | C=N symmetric stretch |
| 941 | Ring breath (side-chain containing S‒S) |
| 805 | Ring breath (main-chain hexahydric-ring) |
| 671 | C‒S stretch |
| 515  483 | S‒S stretch  S‒S stretch |

**Table S4. The assignments of Raman peaks of In_x_-SPAN.**

| **Raman shift (cm^-1^)** | **assignments** |
| --- | --- |
| 174 | C‒S |
| 307 | C‒S in plane bending |
| 367 | C‒S |
| 470 | S‒S |
| 928 | Ring stretch (containing S‒S) |
| 1315 | D band |
| 1542 | G band |

**Table S5. EXAFS fitting parameters at the In K-edge for various samples（Ѕ_0_^2^=0.84 for In）**

| Sample | Shell | CN | R(Å) | σ^2^(Å^2^) | ΔE_0_(eV) | R factor |
| --- | --- | --- | --- | --- | --- | --- |
| In foil | In‒In | 8 | 3.20±0.05 | 0.018±0.003 | 1.11±0.50 | 0.003 |
|  | In‒In | 4 | 3.39±0.017 | 0.018±0.004 |  |  |
| In_2_O_3_ | In‒O | 7.7±0.4 | 2.16±0.03 | 0.006±0.0008 | 2.45±0.61 | 0.009 |
|  | In‒O‒In | 3.7±0.3 | 3.35±0.002 | 0.005±0.0005 | 1.53±1.20 |  |
|  | In‒In | 2.6±0.5 | 3.83±0.017 | 0.005±0.001 |  |  |
| In_2_S_3_ | In‒S | 5.8±0.6 | 2.54±0.07 | 0.012±0.0012 | 3.37±0.99 | 0.01 |
| In_5_-SPAN | In‒S | 4.1±0.5 | 2.46±0.012 | 0.01±0.002 | 3.04±0.77 | 0.003 |
|  | In‒S | 3.4±0.4 | 2.60±0.017 |  |  |  |

CN is the coordination number; R indicates the distance between the inner absorber and its surrounding backscatter atoms; σ^2^ represents the Debye-Waller factor to account for the thermal and structural disorders; ΔE_0_ is edge-energy shift; R factor indicates the goodness of the fit. S_0_^2^ was fixed to 0.84 for all samples, according to the experimental EXAFS fit of In foil.

**Table S6. EXAFS fitting parameters at the In K-edge for In foil and In_5_-SPAN samples（Ѕ_0_^2^=0.78 for In）**

| Sample | Shell | CN | R(Å) | σ^2^(Å^2^) | ΔE_0_(eV) | R factor |
| --- | --- | --- | --- | --- | --- | --- |
| In foil | In‒In | 8 | 3.18±0.059 | 0.018±0.009 | 0.18±0.30 | 0.002 |
|  | In‒In | 4 | 3.39±0.017 | 0.018±0.002 |  |  |
| D-1.0 V | In-S | 2.5±0.4 | 2.48±0.01 | 0.02±0.004 | -1.9±1.8 | 0.019 |
|  | In-In | 3.4±0.5 | 3.22±0.02 | 0.018±0.002 | -0.2±0.99 |  |
| C-3.0V | In-S | 5.1±0.6 | 2.50±0.02 | 0.016±0.003 | 0.47±0.92 | 0.011 |

CN is the coordination number; R indicates the distance between the inner absorber and its surrounding backscatter atoms; σ^2^ represents the Debye-Waller factor to account for the thermal and structural disorders; ΔE_0_ is edge-energy shift; R factor indicates the goodness of the fit. S_0_^2^ was fixed to 0.84 for all samples, according to the experimental EXAFS fit of In foil.

**Table S7. The performance comparisons of various reported SPAN materials. (All mass loading and capacity data are calculated based on the mass of SPAN)**

| **Materials** | **Preparation methods** | **Active**  **material content**  **(wt.%)** | **Mass loading**  **(mg_span_ cm^-2^)** | **Specific capacity**  **(mAh g_span_^-1^)** | **Cycle number (n)** |
| --- | --- | --- | --- | --- | --- |
| **In_5_-SPAN**  **(This work)** | **Electrospinning + Sulfurization** | **47.4** | **8.7** | **542** | **80 (0.5 A g^-1^)** |
|  |  | **47.4** | **5.7** | **609** | **140 (0.5 A g^-1^)** |
| Te_0.052_S_0.948_PAN NS^3^ | Recrystallization + Co-thermal treatment + Sulfurization | 43.2 | 5.8 | 486 | 80 (0.2 A g^-1^) |
| Se_0.071_S_0.929_PAN NS^3^ |  | 43.1 | 5.8 | 446 | 80 (0.2 A g^-1^) |
| CoS_2_-SPAN-CNT^4^ | Electrospinning + Co-precipitation + Sulfurization | 38.5 | 11.9 | 353 | 50 (0.2 C) |
| Co_10_-SPAN-CNT^5^ | Electrospinning + Sulfurization | 38.7 | 6.5 | 400 | 50 (0.2 C) |
| Se_0.38_S_0.62_@pPAN-450℃, treated Li foil^6^ | Co-thermal treatment + Sulfurization | 54.7 | 8.2 | 317 | 200 (0.2 C) |
| SPAN PPY-1%^7^ | Electrospinning + Sulfurization | 43.3 | 6.9 | 461 | 160 (0.2 C) |
|  |  | 43.3 | 9.2 | 361 | 160 (0.2 C) |
| CoSe_2_-6@SPAN^8^ | Hydrothermal + Electrospinning + Sulfurization | 47.5 | 3.6 | 424 | 100 (0.2 C) |
| CNT_1_-SPAN^9^ | Phase-inversion + Sulfurization | 29 | 13.8 | 316 | 100 (0.1 C) |
|  |  | 29 | 17.2 | 312 | 50 (0.1 C) |
| S/PAN/Mg_0.6_Ni_0.4_O^10^ | Ball-milling + Sulfurization | 38.5 | 4 | 470 | 100 (0.1 C) |
| Te_0.04_S_0.96_@pPAN^11^ | Co-thermal treatment + Sulfurization | 47.6 | 7.4 | 414 | 100 (0.2 A g^-1^) |

**Table S8. Recently reported materials of Li-S batteries**

| **Materials** | **Preparation**  **methods** | **Mass loading (mg_S_ cm^-2^)** | **Reversible capacity**  **(mAh g_S_^-1^)** | **capacity retention** |
| --- | --- | --- | --- | --- |
| **In_5_-SPAN-450**  **(This work)** | **Electrospinning + Sulfurization** | **1.5** | **1078**  **(1 A g^-1^)** | **92.1% (1000)** |
| Co-N_3_S_1_  /SeSPAN^12^ | Co-precipitation + Pyrolysis + Electrospinning + Sulfurization | 1.5-2.0 | 866  (1 C) | 99.2% (1000) |
| ZnS/SPAN^13^ | Electrospinning + Sulfurization | 1.3 | 788  (1 A g^-1^) | / (200) |
| NiS_2_/NiSe_2_  @NC/S^14^ | Solvothermal +Sulfurization/Selenization + Melt-infiltration | 2 | 882  (1 C) | 87% (500) |
| Fe_3_O_4_/FeP heterostructure^15^ | Hydrothermal + Carbonization + Phosphorization + Melting-diffusion | 1.0-1.2 | 1093  (1 C) | 62.2% (300) |
| S/(Sn:SnO_2_)  @GO^16^ | Carbothermic reduction + Melting-diffusion | 1.5 | 890  (1 C) | 81% (500) |
| TiO_2-x_^17^ | Electrospinning + Carbonization + Dissolution-recrystallization | 2.0 | 787  (0.5 C) | 74.1% (300) |
| PPc/CE_6_-CNT^18^ | Solvothermal polymerization + Dissolution-recrystallization | 1.5 | 694  (1 C) | 64.5% (500) |
| Co-WO_2_^19^ | Coprecipitation + Carbonization + Melting-diffusion | 1.0 | 1036  (1 C) | 62% (1000) |

**References**

[1] Zhang, H.; Zhang, Y.; Cao, C.; Zhao, W.; Huang, K.; Zhang, Y.; Shen, Y.; Li, Z.; Huang, Y. Lithium–sulfur pouch cells with 99% capacity retention for 1000 cycles. *Energy & Environmental Science* **2024**, *17* (19), 7047-7057.

[2] Chen, J.; Lu, H.; Zhang, X.; Zhang, Y.; Yang, J. Electrochemical polymerization of nonflammable electrolyte enabling fast-charging lithium-sulfur battery. *Energy Storage Materials* **2022**, *50*, 387–394.

[3] Wang, K.; Zhao, T.; Liu, Y.; Yu, T.; Chen, G.; Tang, W.; Li, L.; Wu, F.; Chen, R. Accelerating redox kinetics of sulfurized polyacrylonitrile nanosheets by trace doping of element. *Chemical Engineering Journal* **2024**, *487*, 150300, Article. DOI: 10.1016/j.cej.2024.150300.

[4] Razzaq, A. A.; Yuan, X.; Chen, Y.; Hu, J.; Mu, Q.; Ma, Y.; Zhao, X.; Miao, L.; Ahn, J.-H.; Peng, Y.; et al. Anchoring MOF-derived CoS_2_ on sulfurized polyacrylonitrile nanofibers for high areal capacity lithium-sulfur batteries. *Journal of Materials Chemistry A* **2020**, *8* (3), 1298-1306, Article; Proceedings Paper. DOI: 10.1039/c9ta11390h.

[5] Razzaq, A. A.; Chen, G.; Zhao, X.; Yuan, X.; Hu, J.; Li, Z.; Chen, Y.; Xu, J.; Shah, R.; Zhong, J.; et al. Cobalt coordination with pyridines in sulfurized polyacrylonitrile cathodes to form conductive pathways and catalytic M-N_4_S sites for accelerated Li-S kinetics. *Journal of Energy Chemistry* **2021**, *61*, 170-178, Article. DOI: 10.1016/j.jechem.2021.01.0122095-4956/.

[6] Zhang, W.; Li, S.; Wang, L.; Wang, X.; Xie, J. Insight into sulfur-rich selenium sulfide/pyrolyzed polyacrylonitrile cathodes for Li-S batteries. *Sustainable Energy & Fuels* **2020**, *4* (7), 3588-3596, Article. DOI: 10.1039/d0se00512f.

[7] Yi, Y. K.; Hai, F.; Guo, J. Y.; Gao, X.; Chen, W. T.; Tian, X. L.; Tang, W.; Hua, W. B.; Li, M. T. Electrochemical enhancement of lithium-ion diffusion in polypyrrole-modified sulfurized polyacrylonitrile nanotubes for solid-to-solid free-standing lithium-sulfur cathodes. *Small* **2023**, *19* (48), 202303781. DOI: 10.1002/smll.202303781.

[8] Xu, Z. Q.; Zou, R.; Liu, W. W.; Liu, G. L.; Cui, Y. S.; Lei, Y. X.; Zheng, Y. W.; Nu, W. J.; Wu, Y. Z.; Gu, B. N.; et al. Design of atomic cobalt selenide-doped sulfurized polyacrylonitrile cathode with enhanced electrochemical kinetics for high performance lithium-SPAN batteries. *Chemical Engineering Journal* **2023**, *471*, 144581. DOI: 10.1016/j.cej.2023.144581.

[9] Hu, X. R.; Jiang, H. L.; Hou, Q.; Yu, M.; Jiang, X. B.; He, G. H.; Li, X. C. Scalable SPAN membrane cathode with high conductivity and hierarchically porous framework for enhanced ion transfer and cycling stability in Li-S batteries. *Acs Materials Letters* **2023**, *5* (8), 2047-2057. DOI: 10.1021/acsmaterialslett.3c00450.

[10] Zhang, Y.; Zhao, Y.; Yermukhambetova, A.; Bakenov, Z.; Chen, P. Ternary sulfur/polyacrylonitrile/Mg_0.6_Ni_0.4_O composite cathodes for high performance lithium/sulfur batteries. *Journal of Materials Chemistry A* **2013**, *1* (2), 295-301, Article. DOI: 10.1039/c2ta00105e.

[11] Li, S.; Han, Z.; Hu, W.; Peng, L.; Yang, J.; Wang, L.; Zhang, Y.; Shan, B.; Xie, J. Manipulating kinetics of sulfurized polyacrylonitrile with tellurium as eutectic accelerator to prevent polysulfide dissolution in lithium-sulfur battery under dissolution-deposition mechanism. *Nano Energy* **2019**, *60*, 153-161, Article. DOI: 10.1016/j.nanoen.2019.03.023.

[12] Liu, H.; Xu, Q.; Zhang, Y.; Luo, G. P.; Han, N.; Liu, H. H.; Zhang, X. X. Enhancing the performance of lithium-sulfur batteries by embedding asymmetric Co-N_3_S_1_ single-atom catalysts into hollow SeSPAN nanofibers. *Chemical Engineering Journal* **2025**, *513*. DOI: 10.1016/j.cej.2025.163084.

[13] Liu, Y.; Li, M. X.; Zabrian, D.; Baek, D. H.; Kim, H. W.; Kim, J. K.; Ahn, J. H. Elevating Lithium and Sodium Storage Performance Through the Synergistic Integration of ZnS and Sulfurized Polyacrylonitrile Hybrid Anode Materials. *Energy & Environmental Materials* **2025**, *8* (4). DOI: 10.1002/eem2.70001.

[14] Huang, C.; Yu, J.; Zhang, C. Y.; Cui, Z. B.; Chen, J. K.; Lai, W. H.; Lei, Y. J.; Nan, B. F.; Lu, X.; He, R.; et al. Electronic Spin Alignment within Homologous NiS_2_/NiSe_2_ Heterostructures to Promote Sulfur Redox Kinetics in Lithium-Sulfur Batteries. *Advanced Materials* **2024**, *36* (25). DOI: 10.1002/adma.202400810.

[15] Li, J. H.; Wang, Z. Y.; Shi, K. X.; Wu, Y. J.; Huang, W. Z.; Min, Y. G.; Liu, Q. B.; Liang, Z. X. Nanoreactors Encapsulating Built-in Electric Field as a "Bridge" for Li-S Batteries: Directional Migration and Rapid Conversion of Polysulfides. *Advanced Energy Materials* **2024**, *14* (9). DOI: 10.1002/aenm.202303546.

[16] Nguyen, V. P.; Kim, D.; Kang, J.; Jung, W.; Lim, S.; Yim, K.; Lee, S. M. Heterostructured Sn:SnO_2_ Nanodots for High-Performance Li-S Batteries with Kinetics-Enhanced Cathode and Dendrite-Free Anode. *Advanced Functional Materials* **2025**. DOI: 10.1002/adfm.202507991.

[17] Feng, P.; Dong, K.; Xu, Y. L.; Zhang, X.; Jia, H. J.; Prell, H.; Tovar, M.; Manke, I.; Liu, F. Y.; Xiang, H. X.; et al. Efficient and Homogenous Precipitation of Sulfur Within a 3D Electrospun Heterocatalytic Rutile/Anatase TiO_2-x_ Framework in Lithium-Sulfur Batteries. *Advanced Fiber Materials* **2024**, *6* (3), 810-824. DOI: 10.1007/s42765-024-00380-1.

[18] Zhang, X. M.; Chen, Q. X.; Zhang, W. T.; Hu, H. Y.; Wu, H. M.; Xie, Z. T.; He, X.; Niu, Y. L.; Deng, X. M.; Liu, L.; et al. Supramolecularly Confined Catalysis in Polyphthalocyanine-Crown-Ether Frameworks Boosts Sulfur Redox Kinetics. *Angewandte Chemie-International Edition* **2025**. DOI: 10.1002/anie.202507612.

[19] Zhu, R.; Wu, Z. H.; He, C.; Li, S. Q.; Liu, X.; Wu, M.; Wang, M.; Yan, R.; Li, S. Electronegative Co-WO_2_ Interface with Li Pump Effects for Efficient Polysulfide Conversion in High-Performance Li-Sulfur Batteries. *ACS Nano* **2025**. DOI: 10.1021/acsnano.5c07464.
